# Temporal variability of brain–behavior relationships in fine-scale dynamics of edge time series

**DOI:** 10.1162/imag_a_00443

**Published:** 2025-01-23

**Authors:** Sarah A. Cutts, Evgeny J. Chumin, Richard F. Betzel, Olaf Sporns

**Affiliations:** Department of Psychological and Brain Sciences, Indiana University, Bloomington, IN, United States; Program in Neuroscience, Indiana University, Bloomington, IN, United States; Indiana University Network Science Institute, Indiana University, Bloomington, IN, United States; Stark Neurosciences Research Institute, Indiana University School of Medicine, Indianapolis, IN, United States; Department of Radiology and Imaging Sciences, Indiana University School of Medicine, Indianapolis, IN, United States

**Keywords:** time-varying connectivity, brain–behavior associations, functional MRI, resting state

## Abstract

Most work on functional connectivity (FC) in neuroimaging data prefers longer scan sessions or greater subject count to improve reliability of brain–behavior relationships or predictive models. Here, we investigate whether systematically isolating moments in time can improve brain–behavior relationships and outperform full scan data. We assess how behavioral relationships vary over time points that are less visible in full FC based on co-fluctuation amplitude. Additionally, we perform optimizations using a temporal filtering strategy to identify time points that improve brain–behavior relationships. Analyses were performed on resting-state fMRI data of 352 healthy subjects from the Human Connectome Project and across 58 different behavioral measures. Templates were created to select time points with similar patterns of brain activity and optimized for each behavior to maximize brain–behavior relationships from reconstructed functional networks. With 10% of scan data, optimized templates of select behavioral measures achieved greater strength of brain–behavior correlations and greater transfer of behavioral associations between groups of subjects than full FC across multiple cross-validation splits of the dataset. Therefore, selectively filtering time points may allow for development of more targeted FC analyses and increased understanding of how specific moments in time contribute to behavioral prediction.

## 1 Introduction

The link between individual variation in functional connectivity (FC) and behavior is of central interest in human neuroscience, with clear developmental and clinical applications. Such brain–behavior relationships are typically evaluated using FC computed from continuous and extended brain recordings, capturing minutes of time. Longer time frames and multiple scan sessions have been shown to improve reliability of FC (Birn et al., 2013;Cho et al., 2021;Elliott et al., 2019;Noble et al., 2017) and support improved ability to distinguish individuals (e.g., “fingerprinting”) (Finn et al., 2015;Pannunzi et al., 2017). However, individual differences in functional networks have been discovered at shorter timescales within a scan (Cutts et al., 2023;Peña-Gómez et al., 2018;Van De Ville et al., 2021) raising the possibility that brain–behavior associations may be time dependent. Indeed, extensive literature on time-varying functional connectivity reveals that connectivity patterns fluctuate over time and can be related to different behavioral measures (Eichenbaum et al., 2021;Liégeois et al., 2019;Lurie et al., 2020). Such approaches typically analyze changes in time series characteristics (Betzel et al., 2016;Chang & Glover, 2010;Liégeois et al., 2019;Zalesky et al., 2014), transitions or dwell times of derived network states (Allen et al., 2014;Calhoun & Adali, 2016;Vidaurre et al., 2017), instantaneous measures of FC (Liu et al., 2018;Pedersen et al., 2018;Yaesoubi et al., 2018), or most commonly through smaller sliding windows within a scan session (Calhoun & Adali, 2016;Cohen, 2018;Leonardi & Van De Ville, 2015;Lurie et al., 2020;Preti et al., 2017;Sakoğlu et al., 2010). These fluctuations as well as changes in individual differentiation suggest the possibility that brain–behavior relationships may be stronger during different connectivity patterns only visible from specific moments in time within a scan. Here we assess whether the strength and similarity of brain–behavior relationships across groups of subjects can be improved through targeted selection of time points within a scan session.

Selecting or “filtering” the time series for specific connectivity patterns across subjects could enhance the specificity of behavioral associations. Traditional windowing approaches (Calhoun & Adali, 2016;Leonardi & Van De Ville, 2015;Lurie et al., 2020;Preti et al., 2017) compare connectivity from consecutive frame subsets of varying lengths. However, discontinuous moments can also be assessed based on shared properties of the selected frames. As such, a “filter” could be utilized to retrieve similar moments across subjects, independent of temporal alignment. It has already been shown that fluctuations within a scan exhibit differences in cortex-wide connectivity structure that could be retrieved from such filters. For instance,Sporns et al. (2021)show that a resting-state scan is composed of the transient appearance of co-fluctuations (or coordination of activity) within and between different functional systems. This suggests that apparent connection strength between a pair of brain regions changes over time in relation to variations in system-level or global coordination. As such, even strong co-fluctuations between brain regions at a given moment may not always provide information regarding how that pair of regions relates to a behavior of interest. It is unclear whether data from a full scan include patterns of co-fluctuations that dilute brain–behavior correlations or whether separate moments in time better emphasize different behavioral associations. With approaches looking at instantaneous moments within a scan, similar subsets of frames could be reliably retrieved from the time series and examined across individuals for their relationship with behavioral or phenotypic measures of interest. This can be done by isolating frame subsets from the time series based on the similarity between a template and the activity occurring at specific time points. These templates can be developed for specific behaviors or phenotypes to retrieve moments that amplify brain–behavioral relationships with the measure of interest. Targeted selection of time points based on specific behavioral or phenotypic features may provide additional information unseen using the full length of data.

Past work has found evidence of differences between analyses computed on full FC versus targeted selection of frames within a scan. Edge time series (eTS) is one such method that has been used to deconstruct FC into co-fluctuations between brain regions at single time point resolution (Faskowitz et al., 2020). With this approach,Esfahlani et al. (2020)found that only a handful of high-amplitude moments in time (“events”) dominates the signal seen in full FC and these moments carry most of the individual differences traditionally studied (Betzel et al., 2022). Although these “events” contribute the most information toward full FC, they provide only a sparse representation of the whole time series. This suggests that most brain–behavior relationships have been performed on information from those few moments of high co-fluctuation amplitude. Targeted selection of time points irrespective of co-fluctuation amplitude has already been shown to improve the relationship between specific connectivity patterns with behavior as well as reveal additional or unique information from time points with lower representation in full FC. For instance,Cutts et al. (2023)show that subject identifiability (Amico & Goñi, 2018;Finn et al., 2015) can be improved above full FC with multiple selections of discontinuous framesets. Other work has found that cognitive function is best predicted by frames distributed across the time series with varying co-fluctuation amplitudes (Wehrheim et al., 2023). Additionally, binning time points based on co-fluctuation amplitude reveals distinct relationships with behavioral prediction (Sasse et al., 2023) as well as clinical phenotype (Chumin et al., 2024) unseen in full FC. These findings suggest that developing techniques to distinguish which moments in time contribute most toward behavioral relationships could aid in improved behavioral and diagnostic prediction.

Moments with higher co-fluctuation amplitude have also been found to be more similar across subjects compared with frames with lower amplitude (Esfahlani et al., 2020;Sporns et al., 2021). It is unclear the extent to which greater variance of lower amplitude frames might explain additional, largely unexplored relationships with behavioral variance. However, comparing moments between individuals from resting state proves challenging due to fluctuations occurring independently of time-locked stimuli. High co-fluctuation amplitude moments can be systematically retrieved from the time series and compared across individuals due to their similarities in features (Betzel et al., 2022;Esfahlani et al., 2020). Moments with mid to low co-fluctuation amplitude are more diverse and, therefore, harder to compare across individuals. One solution to reliably target lower amplitude frames is to compare moments across subjects with similar patterns of co-fluctuations between brain regions. This information can be derived from the nodal level based on whether two brain regions display similar or dissimilar activity at a given moment in time (Faskowitz et al., 2020). These moments can be retrieved using a “filtering template” to find the time points with activity patterns that best match the template and then perform analyses on a reconstructed component similar to FC from the selected frames (Chumin et al., 2024;Cutts et al., 2023;Sporns et al., 2021). The structure of these templates can additionally be optimized to improve selection of framesets that improve behavioral relationships. Comparing moments across individuals is then based on connectivity derived from cortex-wide patterns of activity rather than the average across longer time periods dominated by high co-fluctuation moments.

In this study, we assessed whether brain–behavior correlations could be improved through targeted selection of time points irrespective of their contribution to standard FC. First, brain–behavior correlations across 58 separate behaviors were compared across levels of co-fluctuation amplitude, where lower levels contribute less information toward full FC. Second, we developed custom templates to select time points with connectivity patterns that maximize behavioral relationships. This involved implementing multiple rounds of a simulated annealing optimization to develop templates that select time points with similar activity and improve brain–behavior correlations for each behavior. Third, we analyzed the temporal properties of the time points selected both across multiple runs of the optimization for each behavior and across behaviors to assess the locality or dispersion of behavioral information across the time series. Ultimately, co-fluctuation amplitudes displayed differences in brain–behavior correlations which varied based on the behavioral variable. Over multiple optimizations, a handful of behaviors revealed that selection of filtered time points improved correlation strength and similarity of behavioral associations across groups above full FC. Associated time points were distributed across co-fluctuation amplitudes, but most selected frames loaded onto higher amplitude moments. The few moments in time that overlapped across multiple behaviors displayed greater co-fluctuation amplitude and similarity to full FC. This suggests that full FC captures most brain–behavior relationships for some behaviors but may be improved for others if time points are selectively chosen based on the behavior of interest.

## 2 Methods

### 2.1 Dataset and acquisition

Analyses were done on data from the Human Connectome Project (HCP;Van Essen et al., 2013). From the 1,200 data release, 352 subjects (52% female, mean age = 28.44 ± 3.77, age range = 22–36 years) were selected based on low subject motion, high level of data quality/completeness, low incidence of artifact removal, and no family relationships (Cole et al., 2021). Participants were instructed to fixate on a cross during the scan session. Data were collected using a 3T Siemens Connectome Skyra with a 32-channel head coil. Four 15-min resting-state fMRI scans were collected from each participant over 2 days with a gradient-echo EPI sequence, TR = 720 ms, TE = 33.1 ms, flip angle = 52, 2-mm isotropic voxel resolution, and multiband factor = 8. After removal of frames from both the start and the end of the scan session, 1,100 time points remained for a scan duration of 14:33 min. Data acquisition and the minimal preprocessing pipeline are described in detail inGlasser et al. (2013).

A selection of 58 behavioral measures (Table 1) were analyzed from a larger set from the 1,200 HCP subject release (Barch et al., 2013;Van Essen et al., 2013) based on their varied distribution across cognitive, social, and emotional measures (Kong et al., 2019;Liégeois et al., 2019). The confounds of age, sex at birth, body mass index (BMI), framewise displacement (FD), and FreeSurfer intracranial volume were regressed separately for each cross-validation training and testing group. One-hundred cross-validations were created in 80/20 subject splits, where each set consisted of 282 randomly selected training subjects and results were analyzed in the remaining 70 testing subjects.

**Table 1.** List of 58 behavioral measures.

**Behavioral Measures**			
**1.** Visual Episodic Memory	**16.** Walking Endurance	**31.** Agreeableness (NEO)	**46.** Fear – Somatic Arousal
**2.** Cognitive Flexibility (DCCS)	**17.** Walking Speed	**32.** Openness (NEO)	**47.** Sadness
**3.** Inhibition (Flanker Task)	**18.** Manual Dexterity	**33.** Conscientiousness (NEO)	**48.** Life Satisfaction
**4.** Fluid Intelligence (PMAT)	**19.** Grip Strength	**34.** Neuroticism (NEO)	**49.** Meaning & Purpose
**5.** Vocabulary (Pronunciation)	**20.** Odor Identification	**35.** Extraversion (NEO)	**50.** Positive Affect
**6.** Vocabulary (Picture Matching)	**21.** Pain Interference Survey	**36.** Emotion Recognition – Total	**51.** Friendship
**7.** Processing Speed	**22.** Taste Intensity	**37.** Emotion Recognition – Angry	**52.** Loneliness
**8.** Delay Discounting	**23.** Contrast Sensitivity	**38.** Emotion Recognition – Fear	**53.** Perceived Hostility
**9.** Spatial Orientation	**24.** Emotional Face Matching	**39.** Emotion Recognition – Happy	**54.** Perceived Rejection
**10.** Sustained Attention – Sensitivity	**25.** Arithmetic	**40.** Emotion Recognition – Neutral	**55.** Emotional Support
**11.** Sustained Attention – Specificity	**26.** Story Comprehension	**41.** Emotion Recognition – Sad	**56.** Instrumental Support
**12.** Verbal Episodic Memory	**27.** Relational Processing	**42.** Anger – Affect	**57.** Perceived Stress
**13.** Working Memory (List Sorting)	**28.** Social Cognition – Random	**43.** Anger – Hostility	**58.** Self-Efficacy
**14.** Cognitive Status (MMSE)	**29.** Social Cognition – Interaction	**44.** Anger – Aggression	
**15.** Sleep Quality (PSQI)	**30.** Working Memory (N-back)	**45.** Fear – Affect	

These measures were selected from the Human Connectome Project dataset to span cognitive, social, and emotional measures (Kong et al., 2019;Liégeois et al., 2019). Additional information, including field names and descriptions, can be found in the HCP data dictionary (Van Essen et al., 2013).

### 2.2 Preprocessing

The HCP functional data were cleaned of signal artifacts with ICA-FIX (Salimi-Khorshidi et al., 2014). Nilearn signal.clean was used to linearly detrend, band-pass filter (0.008–0.08 Hz;Parkes et al., 2018), confound regress, and standardize all functional data. The functional data were then nuisance regressed using the global signal.

Regional time series were obtained using the Schaefer 200 functional parcellation as provided in the fs_LR surface space (Schaefer et al., 2018). Following nuisance regression, functional data were averaged within each regional node per frame to create 200 spatially distinct time series. Each of the 200 nodes was matched to a set of canonical functional networks as described byYeo et al. (2011): visual (VIS), somatomotor (SOM), dorsal attention (DAN), ventral attention (VAN), limbic (LIM), frontoparietal (FP), and default mode (DMN).

### 2.3 Functional connectivity and edge time series

In this study, functional connectivity (FC) is defined as the statistical dependencies between BOLD time series of every pair of brain regions. Pearson correlation is most commonly used to establish the distance between nodal time series and can be calculated as

$$r_{ij}=\frac{1}{T-1}\sum_{t=1}^{T}z_{i}(t)z_{j}(t),\;$$

where T defines the total number of time points(t)and$z_{i}$represents the z-scored value of regioni. Computing this for every pair of regions produces an[N×N]matrix withNnumber of nodes. All analyses in FC and on connectivity components were vectorized to include$K=\frac{N(N-1)}{2}$unique edges.

FC can be deconstructed into a[K×T]matrix of all edges(K)by time(T). This method is referred to as edge time series (eTS) and produces co-fluctuation time series between each pair of nodes (Faskowitz et al., 2020). Essentially, eTS removes the final averaging step from the Pearson correlation, revealing the instantaneous relationships between the activity of node pairs as

$$r_{ij}(t)=z_{i}(t)z_{j}(t),$$

where$r_{ij}(t)$provides a measure of co-fluctuation of regionsiandjat timet. Averaging eTS across time returns standard functional connectivity (FC) and, therefore, can be used to isolate the exact contribution of temporal features that produce FC. eTS was computed for each subject to derive root sum square (RSS) amplitude values at each time point as

$$RSS(t)=\sqrt{\sum_{i,j>i}r_{ij}{\left(t\right)}^{2}},$$

where each time(t)is assigned a value related to strength of global co-fluctuation across all edges(K).

### 2.4 Bipartitions and connectivity components

Removing amplitude from the time series and preserving whether a brain region’s activity is above or below the mean maintain nearly all information utilized in FC (Sporns et al., 2021). Accordingly, each time step comprises a bipartition of brain regions (or nodes) into respective groups displaying activity either above or below their own means. Selection of frames for analysis can then be based on corresponding framewise properties or similarity between a binarized pattern of activations across brain regions with the bipartitions at each moment in time. This provides both a more efficient means of data storage and a computationally expedient alternative to previous time-varying methods. Additionally, this allows for similar selection of time points for comparison across subjects in resting state.

For each subject and run, the z-scored BOLD time series was binarized by thresholding at zero (BOLD > 0 = 1 and BOLD < 0 = 0). This removed amplitude information while retaining the sign of the signal. An agreement matrix or co-classification matrix (Jeub et al., 2018;Lancichinetti & Fortunato, 2012) provides an account of the frequency in which two nodes are assigned to the same community. This is compared with a null model of the expected frequency of community identity given random permutations of nodes while keeping the number and size of clusters fixed. The null was computed as

$$P_{null}=\frac{1}{l}\sum_{k=1}^{l}\sum_{s=1}^{C_{k}}\frac{\left|C_{ks}\right|}{N}\frac{\left|C_{ks}\right|-1}{N-1},$$

wherelis the number of samples,$C_{k}$the number of clusters in the k-th sample,$\left|C_{ks}\right|$is the number of nodes in a given cluster of$C_{k}$, andNis the total number of nodes. Here each time point(t)was considered as a sample(k)and each bipartition group as a cluster(s). The null was subtracted from the agreement matrix prior to further analysis. Computing an agreement matrix across the bipartitioned time series produces an[N×N]matrix across the selected subset of frames that is nearly identical to standard FC (Sporns et al., 2021). Agreement components (AGc) were created by computing an agreement matrix from subsets of frames within a scan session.

### 2.5 Amplitude binning and frame filtering

Connectivity components allow for network-based analysis on non-continuous moments in time that reflect exact temporal contributions toward FC. Frames can be selectively filtered across time based on multiple criteria of interest (refer toCutts et al., 2023). Two filtering methods were deployed: (1) Binning of frames based on a framewise metric and (2) similarity of frames to a preselected template.

Binning can be performed using any desired criteria of interest that temporally matches the functional time series. Frames can then be sorted into bins of an experimentally chosen size based on the amplitude of the framewise metric. Here, frames were sorted into deciles of RSS amplitude to directly test the appearance of brain–behavior relationships from frames which contribute less toward full FC (Esfahlani et al., 2020).

Template filtering can be performed by computing the similarity between a template and each frame of the time series. Here, distance was computed using the mutual information (MI) between a binarized template (vector of nodal length) and the bipartitioned nodal time series for each time point. MI was computed as

$$MI\left(M,M^{\prime }\right)=\sum_{m\in M}\sum_{m^{\prime }\in M^{\prime }}P\left(m,m^{\prime }\right)log\frac{P\left(m,m^{\prime }\right)}{P\left(m\right)P\left(m^{\prime }\right)},$$

wheremand$m^{\prime }$represent modules within the larger partitions ofMand$M^{\prime }$under comparison.$P\left(m,m^{\prime }\right)$gives the fraction of nodes inmand$m^{\prime }$that are present in both modules (Meilă, 2007). We normalized the measure to account for entropies of bothMand$M^{\prime }$and rescale the unit interval. The top 10% of frames with the highest normalized MI were selected for connectivity components.

MI was selected over other distance measures based on the observation that bipartitions can reconstruct full FC independent of nodal amplitude. A given bipartition and its exact inversion both provide identical information regarding the co-fluctuation of node pairs at a given moment in time. In this case, MI retrieves frames with consistent community labels of the bipartition rather than exact matches to activations. Past work suggests consistency in filtered bipartition frames using templates (Sporns et al., 2021). Further, the use of bipartitions irrespective of amplitude mirrors the strongest instances of similarities between nodes that are the most informative in standard FC analyses. Further information on template filtering can be found (Cutts et al., 2023;Sporns et al., 2021).

### 2.6 Brain–behavior relationships

Irrespective of the filtering or connectivity component approach utilized, results were assessed for cortex-wide behavioral associations separately for each behavior. Spearman correlation was utilized to relate each component edge across subjects to their corresponding regressed behavioral measures. Information was then aggregated across brain–behavior correlation maps by taking the mean absolute value of all edges.

The mean correlational value was used as the cost function in the template optimizations to reduce the complexity of the full brain–behavior matrix into a single value for performance assessment (refer toSection 2.8). This metric was chosen to be more generalizable so that all but the behavioral measures remained constant throughout the optimizations. Greater generalizability was determined based on limited prior assumptions regarding the sign of correlational coefficients or number of expected behaviorally associated edges. There are many other candidate cost functions that could be used to assess improvements in brain–behavior associations. Initial pilot optimizations using metrics such as number of significant correlations (e.g., p < 0.001, p < 0.0001, p < 0.05 Bonferroni corrected) and log of the top percentile of edge p-values performed similarly to the simplified correlational mean implemented in this study.

### 2.7 Multiresolution consensus clustering

The mean brain–behavior correlations found for each RSS decile were clustered into groups of behaviors with similar profiles. A distance metric (here Spearman correlation) was calculated across all pairs of behaviors using their RSS correlation profiles to produce a [behavior x behavior] matrix that was then clustered. Briefly, modularity maximization is a common approach used to compute data-driven communities defined by denser internal connections than would be expected by chance and weaker connections across communities (Esfahlani et al., 2021;Newman & Girvan, 2004). The modularity quality function is computed as

$$Q(\gamma )=\sum_{rs}\left[S_{rs}-\gamma P_{rs}\right]\delta (\sigma_{r},\sigma_{s}),$$

where$S_{rs}$is the similarity between behavior profilesrands,$P_{rs}$is the expected similarity by chance, and$\delta \left(\sigma_{r},\sigma_{s}\right)$is the Kronecker delta function that is equal to 1 when community assignments ofrands, denoted as$\sigma_{r}$and$\sigma_{s}$, are identical and 0 otherwise. The resolution parameterγcontrols the size of communities that modularity detects. The Louvain algorithm (Blondel et al., 2008) was used to maximize the value of Q, as a representation of the quality of community partition, for each entered value of the resolution parameterγ. Here, we used the Potts null model (Traag et al., 2011) and the full FC matrix, including negative edges. We conduct a general “sweep” over theγparameter by sampling at 1,000 different values, followed by a finer sampling within the range where two-community structure appears (10,000 samples). This creates a set of multiresolution communities (Jeub et al., 2018) which are then transformed into a probabilistic agreement matrix showing the fraction of instances in which nodesrandswere assigned to the same communities.

### 2.8 Behavior optimization

An optimization algorithm was used to create custom templates for each behavior that would isolate framesets which maximized brain–behavior correlations across functional connectivity edges. Due to the similarity between the agreement matrix of binarized BOLD time series and full amplitude FC, both binary templates and AGc were utilized for computational efficiency. The algorithm was initiated with randomized binary vectors of lengthN(number of nodes). Normalized mutual information was used to find the similarity between these initial templates and the binarized nodal activity of each time point. The top percentage of best matching frames (in this case 10%) was used to produce AGc for all subjects and runs. Brain–behavior correlations were found for each edge of the average AGc across subjects. In each new iteration, the performance of a template was compared with the template of the previous iteration in order to maximize brain–behavior correlations. If the new template outperformed the previous, it was kept and modified for the next iteration. This process was repeated over 10,000 iterations to allow the algorithm to converge on a stable end state.

With this method, there are 2^N^template permutations which is too large for exhaustive analysis. Therefore, an implementation of the Metropolis-Hastings algorithm (Metropolis et al., 1953) was used to efficiently probe the search space. An objective function D was defined asmean(abs(BB)), whereBBrepresents the brain–behavior correlations for all edges. Improvement upon this objective function was used in conjunction with simulated annealing (Kirkpatrick et al., 1983) to determine the acceptance or rejection of each template iteration. Upon each iteration, a temporary template was created by randomly inverting the binary identities of a handful of nodes from the previous template. The number of altered nodes followed a normal distribution where 1, 2, or 3 nodes were changed with a frequency of 0.68, 0.27, and 0.04, respectively. The success of the temporary template was governed by either the improvement of the objective function or the simulated “temperature” of the system. A cooling schedule was defined as$Temp=T_{0}T_{exp}^{\;\;\;h}$, where$T_{0}$defines the initial temperature,$T_{exp}$the steepness of the gradient decay, andhthe iteration number. An annealing criterion of$e^{-\Delta D/Temp}>R(0,1)$was used to determine acceptance of the current template, whereTemprefers to the simulated “temperature” governed by the cooling schedule andR(0,1)is a random number between [0,1]. This annealing criterion occasionally rejects local optima in favor of continued exploration earlier in the optimization process. As the “temperature” decays based on the cooling schedule, the algorithm increasingly accepts optimal solutions as governed by the objective function. One set of parameters was selected for all behaviors to ensure stable convergence across repeated runs of the optimization within a reasonable time.

Optimizations were performed across 100 cross-validations of 80/20 (train/test) groups for each of the 58 behaviors to evaluate consistency across template outputs. The selected framesets were analyzed for temporal consistency across 7 cross-validations for each subject (based on the minimum from the randomized creation of groups). Selected framesets were additionally compared with framewise properties of the time series such as RSS, similarity to full FC, and framewise displacement (FD). Generalizability was tested by applying the optimized templates to the held-out testing subjects for each cross-validation and analyzing the similarity between brain–behavior correlation maps of the training and testing sets.

### 2.9 Temporal analysis

The temporal location of best matching frames for each optimized behavioral template was recorded for each subject and run. This information was used to compare the consistency of iterations of the optimization as well as determine the overlap between time points retrieved from separate behaviors. The consistency across iterations of the optimization was compared with circshifted nulls (using the MATLAB circshift function) of the selected frame indices for each behavior.

Each iteration of the optimization returned 110 best matching frames regardless of overall fit. Plotted as a binary raster, successful convergence of the optimization is expected to display consistent time frames selected per optimization run. The occurrence of these frame selections was summed across the test group results from multiple cross-validations at each moment in time and the frequency of overlap was then assessed for significance against the same number of randomly shifted framesets. The null model was developed by taking the true framesets and randomly shifting them either forward or backward in time separately for each cross-validation. Frames shifted beyond the boundaries were wrapped around to the opposite side of the time course. This procedure preserved the autocorrelation and temporal spacing between frames while varying the amount of overlap between cross-validations. Ten thousand iterations of the null were performed to establish significance in overlap across optimization runs.

The significant time points established from this method were recorded for every behavior, subject, and run. Overlap across behaviors was additionally established by comparing significant moments in time across behaviors separately for each subject and run to 10,000 rounds of circshifted frames. The number of instances of behavioral overlap was tested against the null across all subjects to establish the degree to which behaviors load onto similar or disparate time points. Considering that RSS has been found to drive the signal present in full FC (Betzel et al., 2022;Esfahlani et al., 2020), behavioral overlap was also compared with co-fluctuation amplitude.

## 3 Results

Here, we used a filtering approach to discover how multiple behavioral relationships change across co-fluctuation amplitude and whether brain–behavior correlations could be improved through targeted selection of time points. We analyzed 352 subjects from the Human Connectome Project (HCP;Van Essen et al., 2013), selected from the “1200 Subjects” HCP release based on low subject motion and no family relationships (Cole et al., 2021). Four resting-state scans were analyzed from each subject. Fifty-eight different behaviors were selected based on their distribution across cognitive, social, and emotional measures (Table 1;Kong et al., 2019;Liégeois et al., 2019).

**Fig. 1.**
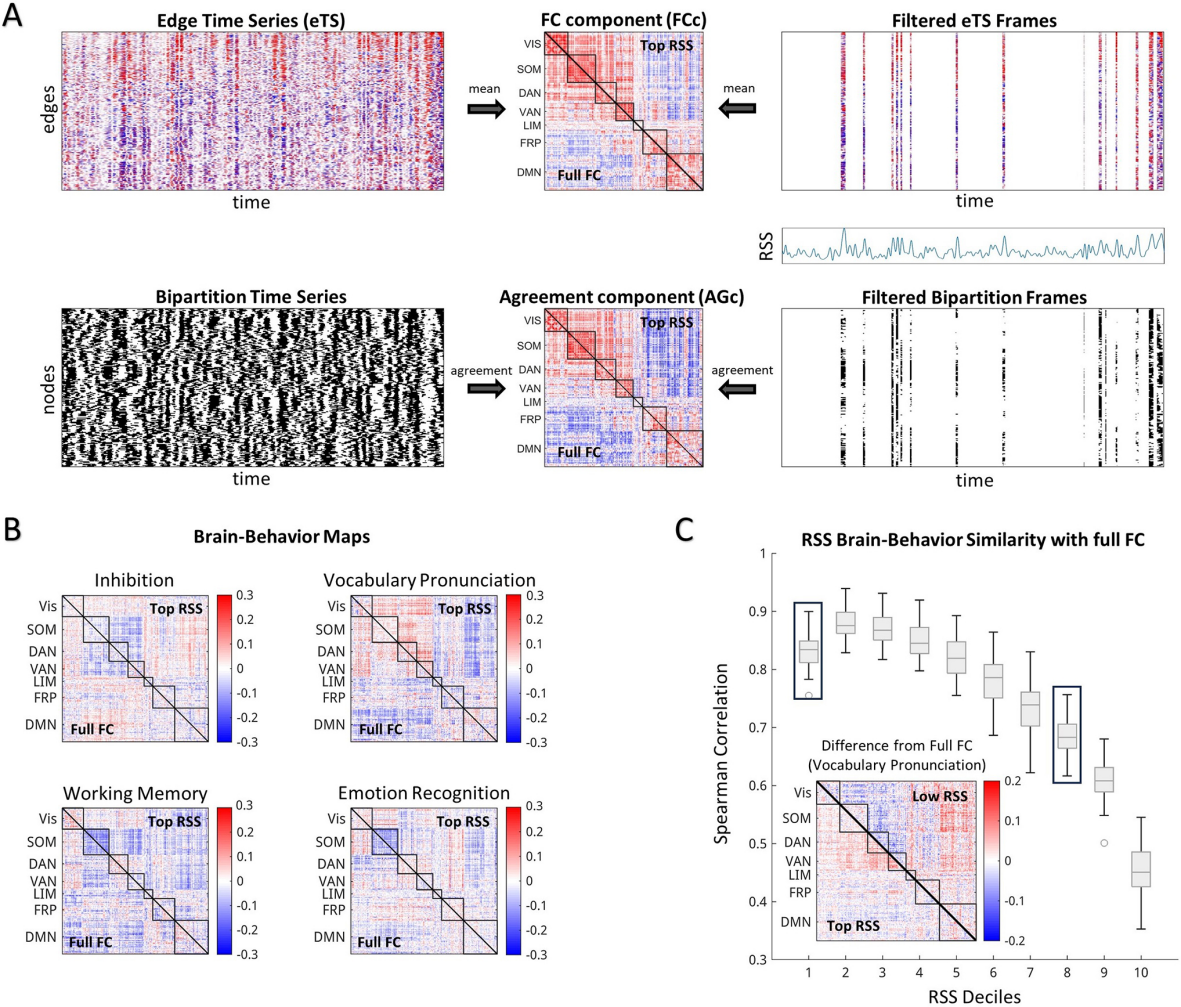
Filtering approach and variability of brain–behavior relationships. (A) Connectivity components created from frames of high co-fluctuation amplitude are nearly identical to connectivity derived from the whole time series. Edge time series (eTS;Faskowitz et al., 2020) forming an edge by time matrix (*top left*), the mean of which, along time, is the “classic” functional connectivity (FC) matrix. Frames can be selected from eTS based on a framewise characteristic of choice (e.g., top 10% co-fluctuation amplitude (RSS);*top right*) and reconstructed into a functional connectivity component (FCc) by taking the mean of edge co-fluctuations across filtered frames. Binarized (or bipartitioned) time series are created by thresholding the z-scored time series (TS) at 0 to split each frame into two communities for a matrix of node by time bipartitions (*bottom left*;Sporns et al., 2021). The agreement across all bipartitioned frames produces a matrix nearly identical to FC (r = 0.98). The agreement across top RSS filtered bipartition frames returns an agreement component (AGc;*bottom right*). The top 10% of co-fluctuation frames capture most of the information in full FC (Esfahlani et al., 2020), with a high similarity between full FC with top RSS FCc (r = 0.94) and top RSS AGc (r = 0.89). (B) Brain–behavior maps created from correlating FCc edge values across subjects with four example behaviors. (C) Similarity between full FC and RSS filtered FCc brain–behavior maps across all behaviors. Boxplots display the median and interquartile range for all behaviors. The inset displays the difference between the behavior map for the example behavior, vocabulary pronunciation, and full FC for the first (Top RSS) and the eighth (Low RSS) deciles.

Connectivity components similar in structure to standard FC were reconstructed using selections of frame subsets. When assessing the full time series, computed co-fluctuations between pairs of brain regions in eTS (Fig. 1A,*top left*;Faskowitz et al., 2020) can be averaged across time to exactly reconstruct FC. Similarly, FC can be nearly reconstructed by binarizing the z-scored time series (“bipartitions”;Sporns et al., 2021) for each brain region and then computing the agreement of partition identity between pairs of brain regions across all bipartitioned frames (Fig. 1A,*bottom left*). Accordingly, disparate frames can be selected based on a property of interest and reconstructed into a connectivity component either through averaging across selected eTS frames or through the agreement of bipartitions. In the case of the highest co-fluctuation time points, this reconstruction from only 10% of the time series greatly resembles full FC (Fig. 1A,*right*). When this handful of high co-fluctuation amplitude frames is selected across subjects, the corresponding brain–behavior correlations across edges greatly resemble behavioral relationships from full FC (Fig. 1B). Similarity between brain–behavior relationships of full FC and components derived from co-fluctuation amplitude likewise systematically decrease with amplitude across all behaviors, suggesting that lower amplitude frames reveal differences in behavioral relationships (Fig. 1C). These differences in RSS bins from full FC can be seen in their varying associations with behavioral measures, as shown for both high- and low-amplitude frames (Fig. 1C,*Inset*;Supplementary Fig. S1). Components created from the agreement across bipartitions (AGc) were used throughout the following analyses for computational efficiency and direct comparison across binning and optimization results (Chumin et al., 2024;Cutts et al., 2023;Sporns et al., 2021).

**Fig. 2.**
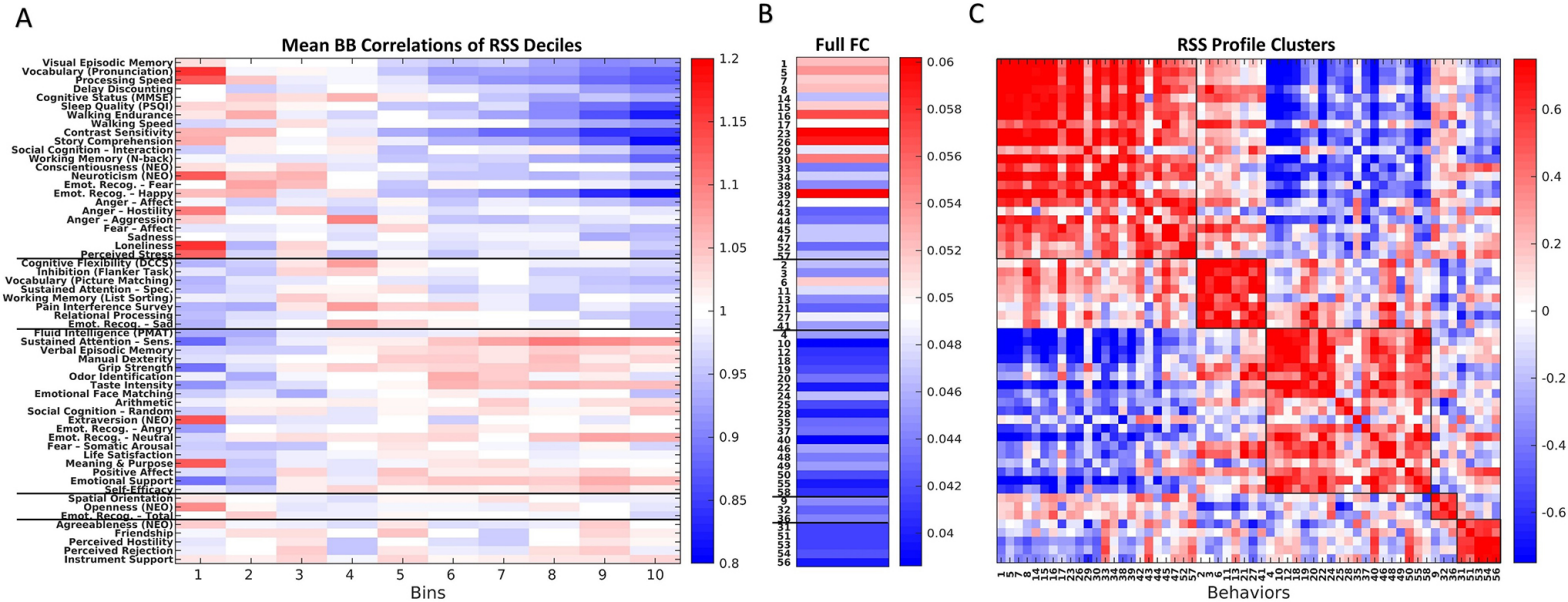
RSS decile binning and community partition of 58 behavioral correlation profiles. Across all panels, behaviors have been ordered based on the RSS clusters defined in panel C. (A) Vectorized decile bins used in the clustering algorithm. The time series was divided into 10 subsets of frames based on RSS amplitude and here average brain–behavior correlations were recorded for each behavior and bin then compared with results from 100 randomly sorted bins. RSS bins were ordered from highest RSS (bin 1) to lowest (bin 10). (B) Mean brain–behavior correlations across all edges as seen in full FC for each behavior and ordered based on derived community identity. (C) A similarity matrix of RSS profiles (from panel A) was clustered into communities using a variant of multiresolution consensus clustering (refer toSection 2) to create groups with denser connections (similarities) within communities than between.

First, behaviors were analyzed across co-fluctuation amplitudes to assess whether lower amplitude frames with less contribution toward full FC displayed unique behavioral associations. Co-fluctuation amplitude varies across time and is computed as the root sum square (RSS) across co-fluctuations between all pairs of brain regions at each time step (refer toSection 2). We created RSS profiles for each behavior to provide a general indication of which amplitude frames of the time series express stronger brain–behavior correlations. Clustering was then used to assess which behaviors loaded onto similar time points within a scan, based on their “preference” for different parts of the RSS amplitude spectrum. We sorted the frames into decile bins based on the RSS strength to assess their relationship with behavioral correlations for each bin. AGcs were created for each RSS decile bin for every subject and averaged across runs. Correlations were computed between each of the 58 behavioral measures and all AGc edges. Strength of brain–behavior correlation was assessed using the mean absolute correlation across all edges. Each behavior consisted of a mean behavioral correlation score for each RSS decile and was compared with 100 AGc made from randomly selected frames. This provided a general mapping of a behavior’s loading onto RSS amplitude (Fig. 2), which were found to cluster behaviors into five groups related to RSS profiles (Fig. 2C; refer toSection 2). These RSS clusters reveal that there are separate groups of behaviors for which there are stronger brain–behavior correlations at different time points. Additionally, a behavior’s loading onto RSS (Fig. 2A) relates to the strength of behavioral correlations captured in full FC. Here, behaviors that mainly load onto higher amplitude frames (found in the first and second clusters) have a slight increased presence in full FC (Fig. 2B) than behaviors that load onto intermediate- to low-amplitude frames (p = 0.0227, independent sample t-test). This suggests that behaviors that load onto lower amplitude frames may be partially obscured in analyses utilizing full FC.

**Fig. 3.**
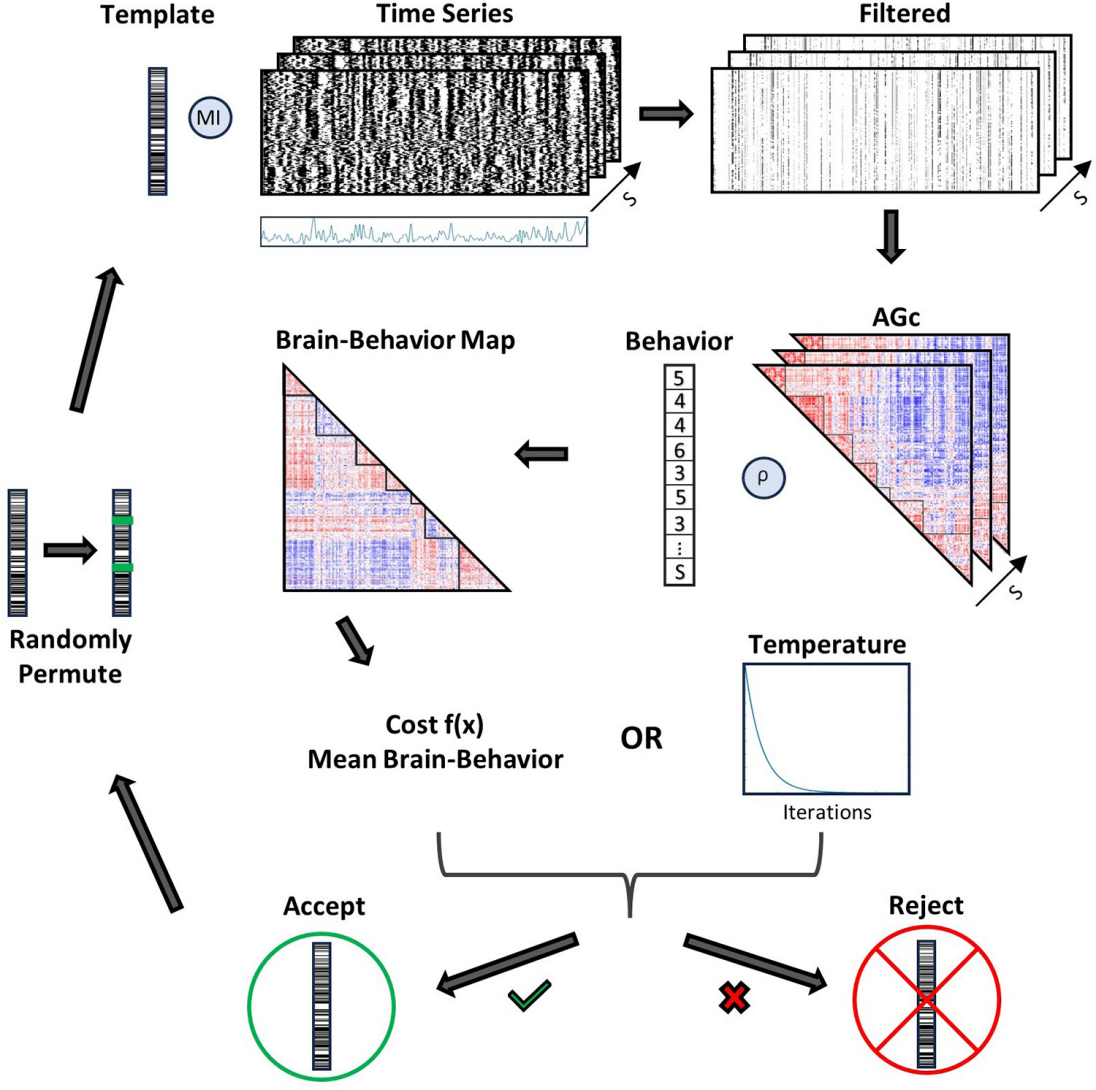
Optimization pipeline utilizing filtering approach. The optimization is initialized with a random vector of node length that is adjusted across each of 10,000 iterations to maximize behavioral relationships. Normalized mutual information (MI) is used to compare the current template to each time step of the bipartitioned time series. The top 10% (110) time points ofT=1,100with highest MI were selected from the time series. An AGc was created from the selected frames and each edge was correlated to behavioral scores across subjects using Spearman’s ρ. This produced a node-by-node brain–behavior correlation map for the selected template that is translated into a cost function (here mean of the absolute value of all edges in the behavior correlation map). The cost function is compared with the previous best solution and is either accepted if performance improves or rejected if the “temperature” is high enough to dictate random exclusion of successful results. Between 1 and 3 nodes of accepted templates are randomly permuted and previous steps are repeated for each iteration.

Second, templates were created to filter time points that improved behavioral relationships across multiple optimizations using simulated annealing. Templates consisted of a binary nodal vector that was used to filter frames based on the similarity (mutual information) between the bipartition of nodes at each time step and the template. This approach allows for the analysis of similar frames across subjects irrespective of timing during resting state. The structure of the binary nodal templates was optimized to provide the strongest behavioral correlations from the top percentage of filtered frames. Here, 10% of similar frames were selected to reduce the effects of artifactual or spurious frames on the AGc. One-hundred optimizations were performed separately for the 58 behaviors where each iteration created a filtering template from 80% of subjects (282 training set) and analyzed results from the corresponding template on the remaining 20% of individuals (70 testing set). A new randomized training and testing set was used for each of the 100 cross-validations. Each optimization was initialized with a random template that was used to filter the top 10% of similar frames from all scans of each subject (Fig. 3). Filtered frames were aggregated into an AGc for each subject across runs and used to create an edgewise brain–behavior correlation map. The strength of behavior correlations from this map was used as the cost function to create filtering templates across multiple optimizations. Each step of the optimization accepts or rejects the current template based on the strength of behavior correlations. Accepted templates are permuted at randomly selected nodes and reintroduced to start a new cycle of the optimization. The end result was an optimized template that selects moments in time for which the cost function is high. This procedure was performed separately to produce unique optimized templates for each of the 58 different behaviors.

**Fig. 4.**
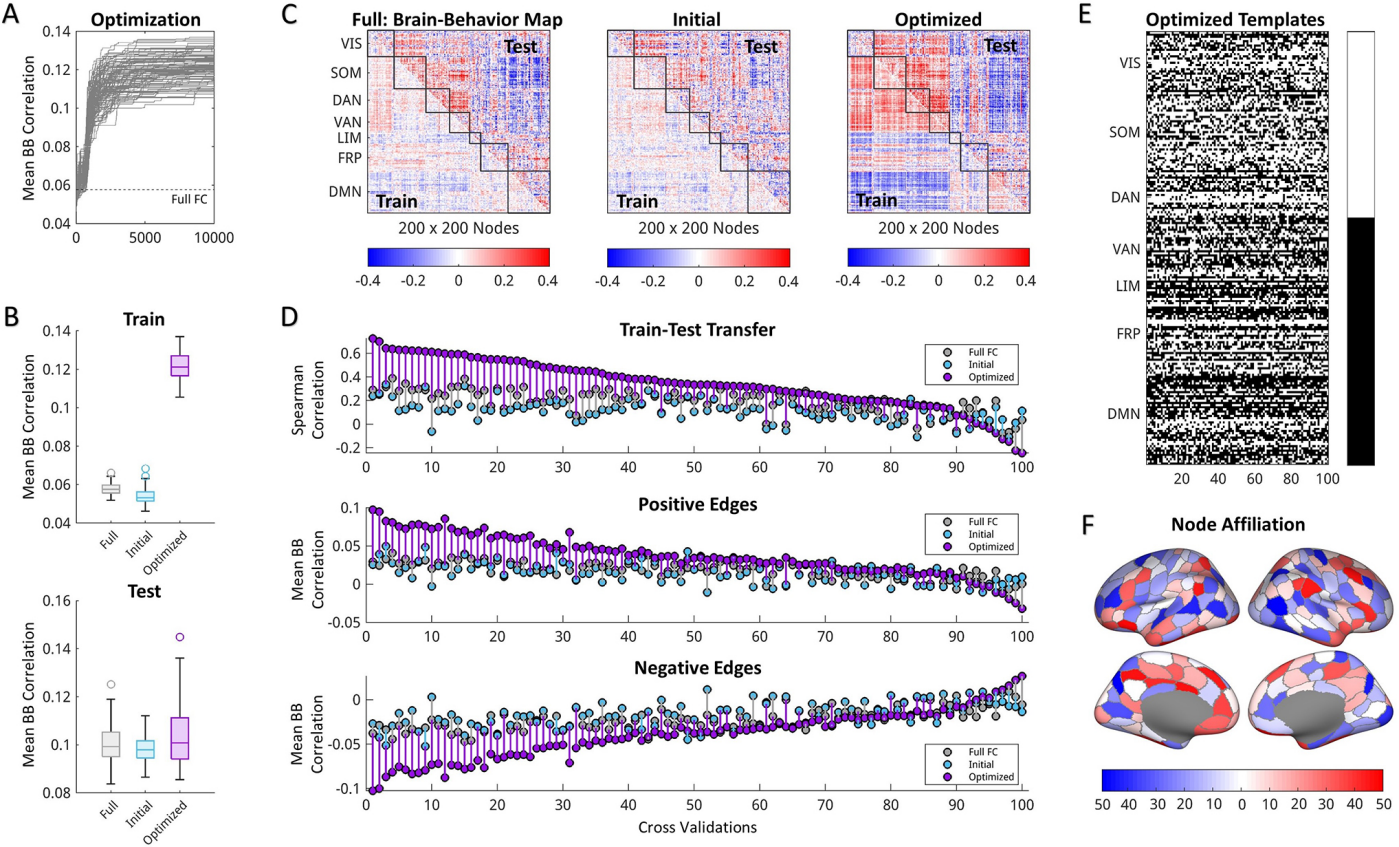
Optimization results for example behavior (vocabulary pronunciation). (A) Mean absolute brain–behavior correlations (mean BB) across each iteration of all cross-validations compared with full FC. (B) Performance in mean BB for brain–behavior correlation maps created from full FC, initial templates, and optimized templates for training (*top*) and testing (*bottom*) groups across all cross-validations. Boxplots display the median and interquartile range for all cross-validations of vocabulary pronunciation. (C) Example brain–behavior correlation maps from full FC, initial, and optimized templates from best performing cross-validation for training (lower diagonal) and testing (upper diagonal) subjects. (D) Performance of full FC, initial, and optimized templates assessed for each cross-validation using Spearman correlation between training and testing brain–behavior maps (*top*) as well as mean BB of positive edges (*middle*) and negative edges (*bottom*). Positive and negative edge results are shown for the testing group using edge masks selected from brain–behavior correlation maps of training subjects and were ordered based on performance of train–test transfer (*top*). (E) Optimized bipartition templates for each of the 100 cross-validations, rectified to align community labels across cross-validations and ordered by functional systems. Templates were further down sampled to display dominant community affiliation for functional systems (*right*). (F) Frequency of community identities assigned to each node from E. Red and blue refer to counts of node affiliation to each template community across cross-validations, where lighter colors and white refer to near chance assignments.

The optimization results from a high performing example behavior (vocabulary pronunciation) are shown inFigure 4(for an additional example behavior, seeSupplementary Fig. S2).Figure 4Ashows the overall strength of behavior correlations across each iteration for 100 cross-validations of the training subjects. As expected, the optimization succeeded in constructing AGcs with much higher behavioral correlations compared with full FC.Figure 4Bshows that the optimized behavior templates filter greater behavioral correlations compared with the initial template as well as full FC for the training subjects. Filters applied to held-out test subjects maintained a similar average of the aggregate measure of brain–behavior correlation (Fig. 4B,*bottom*), but did show a significant increase in correlation strength from each cross-validation over full FC for this given behavior (p < 10^-3^, paired-sample t-test; effect size, d = 0.31). The mean behavioral correlations were reported given their use as the cost function in the optimizations. However, other features of the correlation maps were modified upon optimization that were not readily captured through the average. Additional analyses of the edge correlation distributions, such as kurtosis, were also compared across conditions to better determine how behavioral correlations differed after optimization (Supplementary Figs. S3 and S4). These results suggest that a greater number of edges revealed stronger behavioral correlation following optimization, despite small differences in the averaged measure.

Notably, the brain–behavior correlation maps were also significantly more similar across training and testing subjects.Figure 4Cshows the brain–behavior correlation map from the best performing cross-validation for full FC, the initial template, and optimized frames. The optimized results reveal a much stronger correspondence in behavioral relationships between training and testing groups (additional examples inSupplementary Figs. S5 and S6). This improved train–test transfer (based on Spearman correlation between brain–behavior maps) can be seen across multiple cross-validations and shows significant improvements in similarity across groups (ρ = 0.33) over full FC (ρ = 0.19; p < 10^-15^, paired-sample t-test, effect size, d = 0.90;Fig. 4D,*top*). Positive and negative edges from the training set were compared separately with corresponding edges in the testing set. The mean correlation strength of positive edges (Fig. 4D,*middle*) and negative edges (Fig. 4D,*bottom*) was significantly greater than initial templates (p < 10^-15^, paired-sample t-tests) and full FC (p < 10^-13^, paired-sample t-tests). Similar results were found when the number of filtered frames varied (Supplementary Figs. S7 and S8). This shows that filtering frames using optimized templates allowed greater similarity of brain–behavior correlations across training and testing groups as well as improved mean correlation strength for positive and negative edges.

The optimizations produce consistent template structures that provide additional information regarding which nodes and functional systems are selected for specific behaviors.Figure 4Edisplays the optimized templates for each of the cross-validations ordered based on functional systems. Here, aggregating bipartition community identity across all nodes provides the dominant nodal affiliation for each system (Fig. 4E,*right*).Figure 4Fdisplays the same information as inFigure 4Ebut as the frequency of nodal affiliation with each bipartition community across cross-validations. Interpretation of these templates is dependent on relating bipartitions back to their relationships with functional properties. For instance, all nodes in a template are selected for when using bipartition templates, but some nodes are consistently assigned in alignment or counter to other nodes. These results reveal that there is consistency within the template structures retrieved from these optimizations that can be related back to nodal and network-level features.

**Fig. 5.**
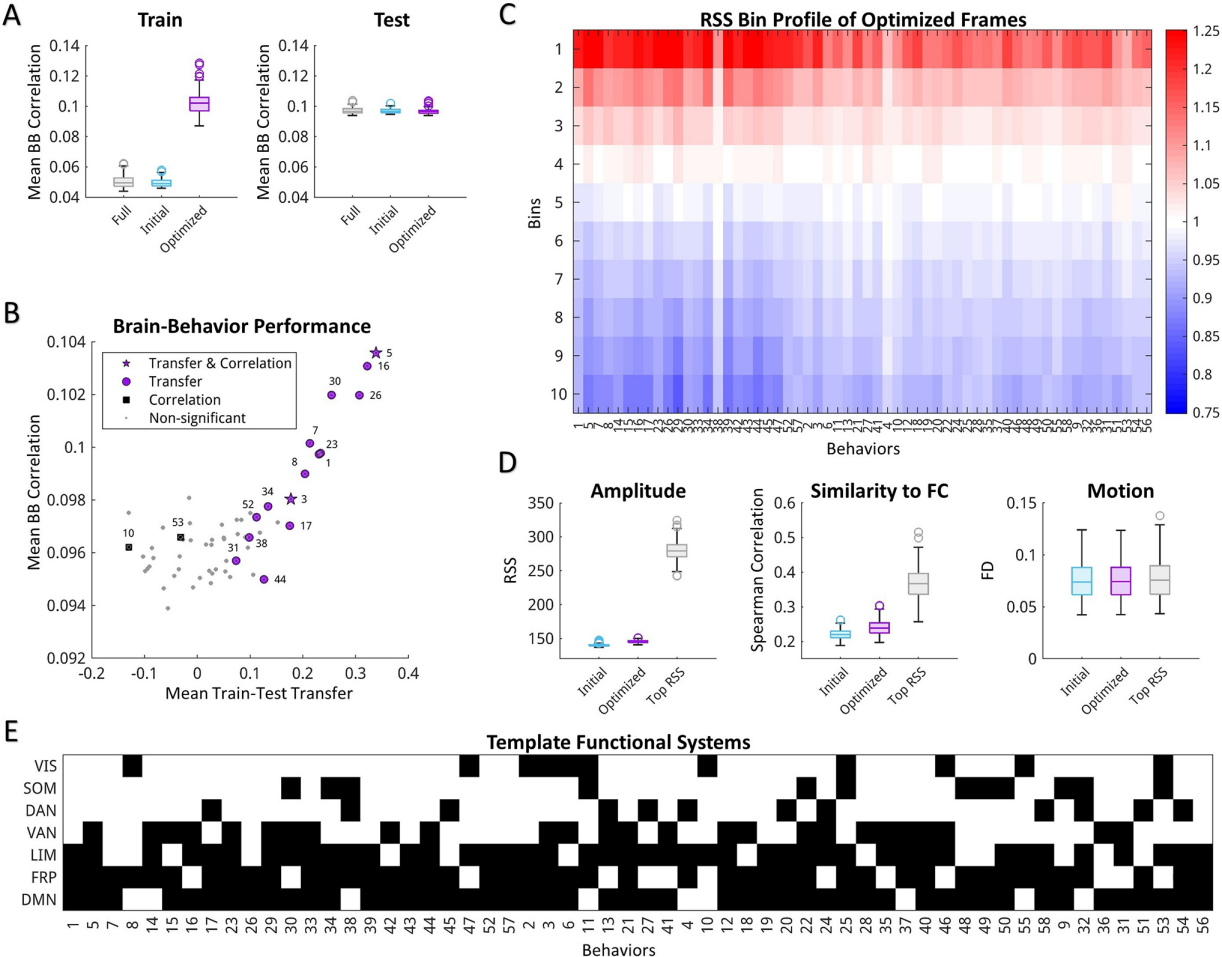
Optimization summary across all behaviors. (A) Mean brain–behavior correlations for every behavior using full FC, initial, and optimized templates for training (*left*) and testing (*right*) groups. Boxplots display the median and interquartile range for all behaviors, averaged within behaviors across cross-validation. (B) All 58 behaviors plotted as strength of behavior correlations of testing subjects versus train–test transfer (mean Spearman correlation between training and testing group brain–behavior maps). Across all cross-validations, each behavior was compared with full FC for train–test transfer, strength of behavior correlation of all edges, and strength of behavior correlations of positive and negative edge sets. Some behaviors showed significant improvements (p < 0.01) over full FC in all mentioned categories (star), improvements in train–test transfer (circle), and mean strength of all correlations (square). Behaviors labeled in purple additionally showed greater strength of mean correlations both in positive and in negative edges. (C) Frequency of optimized frames assigned to each RSS amplitude decile bin, ordered from highest RSS to lowest RSS. Behaviors ordered based on RSS bin clusters fromFigure 2. (D) Mean characteristics of initial, optimized, and top RSS decile frames across all behaviors for RSS (*left*), similarity to full FC (*middle*), and motion (framewise displacement;*right*). Boxplots display the median and interquartile range for averaged framewise properties of each subject. (E) Templates down sampled to display dominant community affiliation for functional systems as shown inFigure 4Ebut for each behavior. Behaviors are ordered based on RSS clusters inFigure 2.

Figure 5provides a summary of the results across all analyzed behaviors.Figure 5Ashows the average mean behavioral correlation from all cross-validations improved for each behavior in the training set. Similar levels of averaged brain–behavior correlations were found across all conditions of the testing set for most behaviors.Figure 5Bshows the detailed results as displayed inFigure 4, but for all behaviors. Significance values and effect sizes for comparisons between optimized and full FC as well as optimized and initial frames are listed inSupplementary Tables S1 and S2for all behaviors. Here, all behaviors with significantly greater train–test transfer of behavioral correlations compared with full FC (p < 0.01, one-tailed t-test uncorrected) also maintained significantly greater strength of positive and negative edges in the testing set. Similar results for Bonferroni corrected as well as example AGcs and brain–behavior correlation maps across multiple behaviors are displayed inSupplementary Figures S9 and S10. A handful of behaviors also showed both greater transfer and correlation strength or only greater correlation strength than full FC. Additionally, the optimized templates were not interchangeable across behaviors. Optimized templates filtered AGc with higher mean behavioral correlations within behavior than across behaviors (Supplementary Fig. S11) and the templates were notably more similar across cross-validations within behaviors than between behaviors (Supplementary Fig. S12). This suggests that the optimizations were locking onto different features of the time series at separate time points that were not reducible to a single best pattern to describe multiple behaviors. Instead, optimizing individual behaviors repeatedly selected for template patterns specific to those behaviors.

The filtered frames from the optimization were further matched to their corresponding RSS decile bin (shown inFig. 2).Figure 5Crelates the count of optimized frames that belong in each RSS bin to the average binning identities of 100 randomly selected bins. Across all cross-validations of each behavior, optimized templates select for frames with higher RSS and similarity to FC than the initial template without selecting for increased motion artifact based on framewise displacement (Fig. 5D). However, compared with the highest RSS decile bin, optimized frames maintain lower RSS (p = 0, independent sample t-test) and lower similarity to FC (p < 10^-15^, independent sample t-test), suggesting that filtering recruits frames from lower co-fluctuation amplitudes to improve behavioral correlations. These improvements in behavioral correlations and train–test transfer relate to increased variability between subjects. Filtering time points using behavioral templates increased the differentiation of component patterns from full FC (Supplementary Figs. S13 and S14) and subject variability (Supplementary Fig. S15).

Further, the down sampled functional system structures of the templates were computed across all behaviors (Fig. 5E) as shown inFigure 4Eand ordered based on the RSS clusters derived inFigure 2. Most behaviors produced templates with bipartitions separately grouping VIS, SOM, DAN, VAN and LIM, FRP, DMN. This pattern was consistent across behaviors with reliable deviations of select functional systems and largely independent of RSS classification. The similarities in the down sampled network templates likely result from the higher amplitude frames selected for most behaviors. Differences across behaviors and improvements over full FC, however, appear with the addition of lower amplitude frames with differing patterns of co-fluctuations between networks. Further assessment of node co-assignment to the same bipartition communities within optimized templates further shows that most nodes are consistently paired across functional systems rather than within systems (Supplementary Fig. S16). There is little overlap across behavior templates regarding which node pairings are consistently present within the same bipartition community (Supplementary Fig. S17), suggesting that the optimizations are selecting for varied template structures across behaviors. The one notable source of overlap between behaviors, however, is the assignment of nodes from the frontoparietal system to the same bipartition community. This functional network has been associated with higher subject variability and fingerprinting capability (Amico & Goñi, 2018;Finn et al., 2015;Peña-Gómez et al., 2018), supporting the idea that these optimized templates are selecting for moments from the scan with higher intersubject variability related to behavioral measures.

**Fig. 6.**
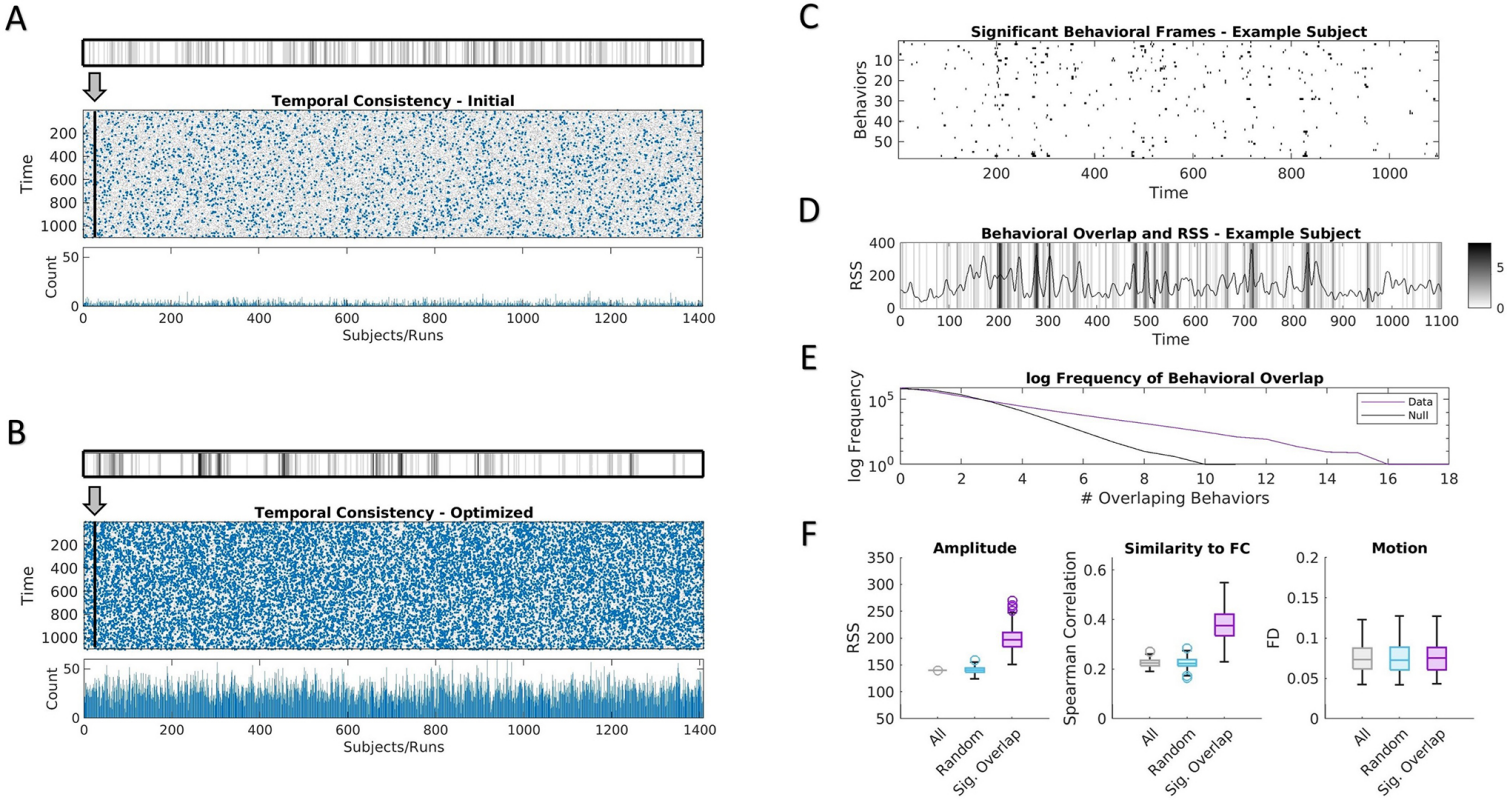
Temporal consistency and behavioral overlap. (A) Temporal consistency of frames filtered using the initial template of example behavior (vocabulary pronunciation) across multiple cross-validations. Results are shown for cross-validation training templates applied to subjects in the testing groups. Frequency of frames selected across all considered cross-validations for each time point is displayed for every subject and run. Example subject shown (*top*) as plotted for every subject and run (*middle*). Time points with significant consistency between cross-validations (p < 0.01; compared with 10,000 circshifted nulls) plotted in blue and the frequency of occurrence of these significant time points are shown (*bottom*). (B) Temporal consistency of optimized templates of example behavior (vocabulary pronunciation). (C) Frames with significant consistency as shown in*B*plotted for each behavior across time for one example subject and run. (D) Overlap across behaviors of significant time points as in*C*for the example subject and run. Corresponding RSS values displayed. (E) Plot of log frequency of instances of behavioral overlap of significant frames across all subjects and runs of the test group compared with distribution of 10,000 circshifted nulls. (F) Mean characteristics of frames across all subjects and runs for all frames, matching number of randomly selected frames, significant behavioral overlap (p < 0.01) for RSS (*left*), similarity to FC (*middle*), and motion (framewise displacement;*right*). Boxplots display the median and interquartile range for averaged framewise properties of each subject.

Third, the temporal properties of time points selected from behavior templates were assessed and compared across behavioral measures. So far, the results have been aggregated over the selected frames of all subjects and runs as well as multiple cross-validations, but the optimization pulls frames from specific time points whose properties can be analyzed directly. Out of the random 80/20 (train/test) splits of the 100 cross-validations, subjects were selected multiple times across both groups. Over multiple cross-validations of an individual subject, the optimizations consistently targeted a select set of specific time frames.Figure 6A(*top*) shows an example heatmap for the frequency of frame selection for the example behavior (vocabulary pronunciation) across seven cross-validations of testing set results for a select subject and run. Each column of the matrix depicted below (Fig. 6A,*middle*) consists of the corresponding heatmaps of each subject and run. Heatmaps of all subjects and runs were compared with 10,000 circular shifted nulls to determine a significance score for each frame (refer toSection 2). Frames were then selected based on a threshold of p < 0.01 (uncorrected) for significantly greater temporal consistency across optimizations than chance. A stricter threshold of p < 0.001 provides similar results (shown inSupplementary Fig. S18and a separate behavior inSupplementary Fig. S19). The number of frames found to be significantly consistent among cross-validations was much higher for the optimized templates (Fig. 6B; mean = 29.02) than for the initial template used at the start of the optimization (Fig. 6A; mean = 3.30; p = 0, paired-sample t-test). This suggests that the optimized templates allowed for the consistent selection of time points in held-out test data despite multiple independent initializations of the optimization with different groupings of subjects.

These significant frames were then compared across behaviors. InFigure 6C, the significant frames are shown for each behavior of an example subject and run. From that, selected frames across behaviors appear to largely occur independently of one another but a handful of frames reveal overlap across behaviors.Figure 6Dshows the behavioral overlap of the example subject and run shown inFigure 6Cand compares the frame selection with the RSS amplitude time series. Across all subjects and runs, there were time points with greater behavioral overlap compared with 10,000 circular shifted nulls (Fig. 6E). The time points with significant behavioral overlap (p < 0.01, uncorrected) were averaged across all runs of each subject and general framewise properties were compared with results from initial and all frames. InFigure 6F, moments in time with significant behavioral overlap display increased RSS amplitude, higher similarity to full FC, and nonsignificant differences in head motion compared with initial templates and all frames.

## 4 Discussion

Functional connectivity is becoming increasingly used toward the development of brain-based biomarkers of healthy cognition, demographics, and clinical diagnosis (Chen et al., 2021,^2022^;Chumin et al., 2024;Dhamala et al., 2021;Dizaji et al., 2021;Dubois et al., 2018;Lake et al., 2019;Parkes et al., 2020;Sripada et al., 2020;Uddin et al., 2013;Wu et al., 2023). There are already several machine learning and predictive models that have been applied toward improving behavioral prediction and developing a deeper understanding of what networks can tell us about cognition and clinical phenotype (Cai et al., 2021;Cui & Gong, 2018;Finn et al., 2015;He et al., 2020,^2022^;Kawahara et al., 2017;Kong et al., 2019,^2021^;Ooi et al., 2022;Pervaiz et al., 2020;Sasse et al., 2023;Shen et al., 2017;Wehrheim et al., 2023). A large portion of FC research has been done on fMRI data, so methodological constraints and the slow hemodynamic response of the BOLD signal (Buxton, 2013;Lake et al., 2020;Logothetis, 2002;Schwalm et al., 2017) have led to the preference of conducting analyses on full scan sessions of 20–40 min of data per subject to improve reliability (Birn et al., 2013;Elliott et al., 2019;Laumann et al., 2015;Noble et al., 2017). Recent studies, however, suggest that additional behavioral information can be obtained from the analysis of temporal fluctuations (Cutts et al., 2023;Eichenbaum et al., 2021;Fong et al., 2019;Liégeois et al., 2019;Lurie et al., 2020;O’Connor et al., 2022;Peña-Gómez et al., 2018;Van De Ville et al., 2021). There are already several approaches within the time-varying literature (Allen et al., 2014;Betzel et al., 2016;Calhoun & Adali, 2016;Chang & Glover, 2010;Cohen, 2018;Eichenbaum et al., 2021;Leonardi & Van De Ville, 2015;Liégeois et al., 2019;Lindquist et al., 2014;Liu et al., 2018;Lurie et al., 2020;Pedersen et al., 2018;Preti et al., 2017;Sakoğlu et al., 2010;Vidaurre et al., 2017;Yaesoubi et al., 2018;Zalesky et al., 2014), but there are few answers regarding whether methods can be applied evenly across specific behaviors or phenotypes of interest. Here, we assess whether behavioral relationships across a suite of behaviors are visible in full FC or whether there are temporal fluctuations within a scan with stronger relationship with behavioral variance. This was done by analyzing whether brain–behavior associations change based on co-fluctuation amplitude and by filtering frames based on specific patterns of activity (bipartitions). We used optimizations to develop bipartition templates to isolate time points consistently across subjects with stronger behavioral associations and determine whether separate moments are better for different behaviors. We also show that the time points filtered for these behaviors are consistent across multiple optimizations and create transferable brain–behavior associations in held-out subjects. We found that a few moments in time improve behavioral associations across multiple behaviors, but that some behavioral information loads onto moments that are less prominent in full FC.

Our approach provides a framework to select behaviorally relevant moments in time from a typical scan session consistently across individuals. These results suggest that moments in time carry different signatures of information that are relevant toward specific brain–behavior associations. We found that there are some moments that are more informationally rich than others and that behavioral relationships differ based on co-fluctuation amplitude as well as ability to be detected from full FC. Moments that enhance behavioral associations differ across behaviors, suggesting that filtering can enhance connectivity patterns based on the phenotype or behavior of interest. This is done with resting state where normally large amounts of data are needed to ensure the researcher is analyzing stable FC across individuals. Rather than analyzing summaries over longer periods of time, finding moments that are similar across subjects in resting state could fulfill this need with less data and clearer results with the added benefit of improved temporal localization of behaviorally related signatures. Recent work suggests that thousands of subjects are needed to establish strong and reliable brain–behavior associations (DeYoung et al., 2022;Liu et al., 2023;Marek et al., 2022;Rosenberg & Finn, 2022). However, filtering time points based on specific behaviors or phenotypes provides an additional avenue for exploring intersubject variability in behavior. Longer scans would still be required to identify enough time points that better relate to specific behaviors, but by sorting frames based on their behavioral relevance, more nuanced information may be achieved over otherwise static full FC.

Across multiple filtering approaches, this study shows that behaviors often load onto similar time points as groups. For instance, clusters of behaviors exhibit similar associations within frames of RSS amplitude ranges (Fig. 2). Additionally, using optimizations, we find that most temporal overlap across behaviors occurs at high amplitude moments (Fig. 6). Although there is a dispersion of behaviorally relevant information in the time series that differs across behaviors, it remains unclear what causes instances of overlap between them. Here, we highlight that behaviors that prefer higher amplitude frames are more likely to be represented in full FC. It remains unclear, however, why behaviors load onto specific amplitude moments and how those that share similar “strategies” relate to each other behaviorally. Future work focusing on specific behaviors would further reveal which aspects of connectivity are highlighted from filtered frames, whether these features are meaningfully related across behaviors, and why certain behaviors load onto lower amplitude moments with less contribution toward the time-averaged full FC.

Linking behavioral and phenotypic associations in FC back to mechanistic or neurobiologically plausible sources will likely require improvements in temporal specificity. Along with fluctuations in time-varying FC (Allen et al., 2014;Betzel et al., 2016;Calhoun & Adali, 2016;Chang & Glover, 2010;Cohen, 2018;Eichenbaum et al., 2021;Leonardi & Van De Ville, 2015;Liégeois et al., 2019;Liu et al., 2018;Lurie et al., 2020;Pedersen et al., 2018;Preti et al., 2017;Sakoğlu et al., 2010;Vidaurre et al., 2017;Yaesoubi et al., 2018;Zalesky et al., 2014), individually distinctive signatures also vary over time (Cutts et al., 2023;Peña-Gómez et al., 2018;Sasse et al., 2023;Van De Ville et al., 2021). As such, the relationship between intersubject variability in FC and behavioral measures will change based on the connectivity pattern compared across subjects. With enough data, FC stabilizes within subjects (Gratton et al., 2020;Laumann et al., 2015;Noble et al., 2017) to provide a clear baseline for intersubject comparisons. However, moments in time that emphasize connections between specific brain regions or functional systems can also provide a scaffold for comparisons that can be related back to intersubject variability of specific behaviors. The origin of these temporal fluctuations in FC remains unclear, with some studies suggesting an origin in neuronal processes (Matsui et al., 2019;Thompson, 2018) or network dynamics (Heitmann & Breakspear, 2018;John et al., 2022), while others interpret FC states as expected stochastic variations around a central tendency that can be observed at the full scan length (Ladwig et al., 2022;Laumann et al., 2017).

Regardless of the origin, finding the moments in time that contribute most toward behavioral associations allows for closer inspection of the relationship with other neurobiological signatures or noise. For instance, out of the 15 behaviors with significant improvements over full FC, 7 were classified as task performance, 4 as self-report, and 4 as unclassified (based onLiégeois et al., 2019). This suggests a potential dichotomy where behaviors perform better either from optimized frame subsets or from full FC, and this could be due to the trait-like nature of the behavior in question or the duration it is expected to occur. Additionally, these improvements in behaviors from temporal filtering might be related to associations with physiological signatures. Although behaviorally filtered frames from this study did not show systematic differences attributable toward noise (such as head motion;Figs. 5Dand6F), and potential confounding variables such as age, sex, and BMI were regressed out, the time points selected for improvements in behavioral correlations could be related to additional physiological or behaviorally relevant time stamps. Some of these improvements in behavioral associations may be driven by variation in physiological signatures typically considered as artifact, but it is important to determine both where such improvements in behavioral associations arise as well as the relationship between potential sources of artifact and their correlations with behavioral or phenotypic traits (He et al., 2022;Uddin, 2020). Additionally, future work will involve optimizing behavioral relationships with movie and task data where the exact stimuli that drive behavior relationships can be assessed through comparison with the contents of the movie or task of interest.

Determining the temporal origin of associations between phenotypic measures and functional networks could provide greater insight into how the brain works and establish more efficient methods for processing neuroimaging data. Aging or clinical cohorts is one area where applying targeted filtering methods could enhance the quality of the data under investigation. Such cohorts are often prone to discomfort or increased movement while in the scanner (Satterthwaite et al., 2019). Using a template filter to recover frames which amplify intersubject differences in a behavioral or phenotypic measure of interest could improve data quality (if such moments are not themselves related to artifact that commonly impacts time-averaged FC). Future work, for instance, assessing differences in template filtered frames might reveal age-related differences not present in standard FC. The widespread application of these methods, however, will require development of phenotypic or clinical-specific templates from representative cohorts and through utilizing domain expertise.

This study provides proof of concept for the selective filtering approach and shows that behaviors load onto disparate time points scattered throughout the scan session. The templates created here were optimized on 58 behaviors selected to represent a larger set (Barch et al., 2013;Van Essen et al., 2013). Although this provides an overview of different characteristics of behavioral measures in FC, templates for each of these behaviors were produced from the same optimization parameters. Although behaviors were assessed equally for balanced comparison, template structure is likely influenced by this assumption. Each of these behaviors would benefit from specialized attention to improve targeted frame selection. For instance, the cost function used in this study aims to improve average brain–behavior correlations across the cortex. Certain behaviors would not be expected to involve cortex-wide associations. Therefore, we suspect this choice in cost function selects high-amplitude moments with greater potential range of edge amplitudes across the cortex to capture behavioral variation. These higher amplitude moments further consist of bipartitions with characteristic decoupling between intrinsic versus task-positive systems (Sporns et al., 2021). The notable differences in behaviors that exceed full FC, therefore, likely relate to the consistent deviations away from this template structure. Further work incorporating spatial considerations into template development could significantly improve the method’s ability to isolate behaviorally relevant moments. We also know that behaviors occur at varying timescales, therefore, optimization procedures would likely benefit from considering the number of selected frames, sequences of frames, or the use of specific nodes or functional networks. Here, analyzed components were created from 10% of the time series and it remains unclear whether behaviors would benefit from varying the quantity of considered frames (results displayed for an example behavior using 5% (Supplementary Fig. S7) and 15% frames (Supplementary Fig. S8)). Ultimately, assessment by domain experts in specific behaviors or phenotypes could further clarify the relationship between the optimized templates and their measures through increased incorporation of expected spatial and temporal features characteristic of the targets of interest.

The structure of the templates themselves is also informative of the features filtered for each behavior and should be investigated further in future work. Here, we show that the templates retrieve consistent patterns within behaviors that are not reducible to a global pattern that best describes all behaviors (Supplementary Figs. S11 and S12). This approach reveals the benefits of optimizing behaviors separately compared with applying all analyses uniformly to a single set of time points. The specificity of behavioral templates suggests potential biological relationships between the nodal structure of the templates and the behavior they were optimized on. This study found that optimized templates display different bipartitions across functional systems (Fig. 5E), but it remains unclear what extent of the template is relevant toward the behavior of interest. Further work is also needed to determine how bipartitions correspond to the literature on specific behaviors. Since bipartitions represent nodes that actively coordinate within but not across communities, exact overlap between templates and known behaviorally relevant activations have yet to be assessed at a time-resolved level. Currently, this makes direct interpretation of the templates challenging regardless of their utility in retrieving desired framesets. That said, future work assessing multiple behaviors simultaneously could lock onto separate time points best related to behavioral constructs. For instance, canonical correlation analysis (CCA) could be utilized to discover time points across multiple brain–behavior relationships simultaneously and factor analysis could be used to derive optimizable scores across behavioral measures. There are many remaining avenues to explore regarding how behavioral templates relate to nodal activations and why specific time points within the scan amplify behavioral associations.

Lastly, the optimization occasionally produces an exact inversion of the brain–behavior map found in most other cross-validations (as seen in the cross-validations with worse train–test transfer inFig. 4D,Supplementary Fig. S6). This likely occurs when the randomly selected subjects in the testing set involve outliers of the overall trend. A similar issue with the optimization is that for most behaviors a handful of cross-validations do no better than the initialized template with low train–test transfer as well as clear lack of functionally relevant structure in edgewise correlations of the brain–behavior map. However, it should be noted that the same cross-validations that experience inversions or fail to optimize clear edgewise correlations also mirror failures or inversions in full FC (Supplementary Fig. S6), likely relating issues back to differences in the groups of subjects analyzed. Overall, successful optimizations repeatedly return the same brain–behavior correlation map as well as similarities in filtered time indices across multiple iterations. Additional work is necessary to determine how these templates lock onto and improve behavioral associations.

In summary, we show that behavioral relationships vary across time and do not always occur at the same moments for different behaviors. These behavioral relationships map onto specific activity (bipartition) templates that can be used to filter similar functional connectivity patterns across subjects even during resting state. These optimized behavioral moments occur across RSS amplitudes and, therefore, may benefit from filtering approaches to emphasize brain–behavior relationships not as visible in full FC. Future applications abound, including studies probing for individual differences that involve developmental or clinical cohorts, and that leverage emerging machine learning and A.I. technology.

## Supplementary Material

Supplementary Material

## Data Availability

Neuroimaging and behavioral data from the Human Connectome Project are available after signing a data use agreement at the link:https://db.humanconnectome.org/. Code for computing edge time series and bipartitions can be accessed athttps://www.brainnetworkslab.com/coderesourcesand additional graph theoretic metrics are found athttps://sites.google.com/site/bctnet/. Code used to perform frame filtering, create connectivity components, and produce optimized templates can be found athttps://github.com/sarahacutts/temporal-behavior-optimization.

## References

[b1] Allen , E. A. , Damaraju , E. , Plis , S. M. , Erhardt , E. B. , Eichele , T. , & Calhoun , V. D. ( 2014 ). Tracking whole-brain connectivity dynamics in the resting state . Cerebral Cortex , 24 ( 3 ), 663 – 676 . 10.1093/cercor/bhs352 23146964 PMC3920766

[b2] Amico , E. , & Goñi , J. ( 2018 ). The quest for identifiability in human functional connectomes . Scientific Reports , 8 ( 1 ), 8254 . 10.1038/s41598-018-25089-1 29844466 PMC5973945

[b3] Barch , D. M. , Burgess , G. C. , Harms , M. P. , Petersen , S. E. , Schlaggar , B. L. , Corbetta , M. , Glasser , M. F. , Curtiss , S. , Dixit , S. , Feldt , C. , Nolan , D. , Bryant , E. , Hartley , T. , Footer , O. , Bjork , J. M. , Poldrack , R. , Smith , S. , Johansen-Berg , H. , Snyder , A. Z. , & Van Essen , D. C. ( 2013 ). Function in the human connectome: Task-fMRI and individual differences in behavior . NeuroImage , 80 , 169 – 189 . 10.1016/j.neuroimage.2013.05.033 23684877 PMC4011498

[b4] Betzel , R. F. , Cutts , S. A. , Greenwell , S. , Faskowitz , J. , & Sporns , O. ( 2022 ). Individualized event structure drives individual differences in whole-brain functional connectivity . NeuroImage , 252 , 118993 . 10.1016/j.neuroimage.2022.118993 35192942

[b5] Betzel , R. F. , Fukushima , M. , He , Y. , Zuo , X.-N. , & Sporns , O. ( 2016 ). Dynamic fluctuations coincide with periods of high and low modularity in resting-state functional brain networks . NeuroImage , 127 , 287 – 297 . 10.1016/j.neuroimage.2015.12.001 26687667 PMC4755785

[b6] Birn , R. M. , Molloy , E. K. , Patriat , R. , Parker , T. , Meier , T. B. , Kirk , G. R. , Nair , V. A. , Meyerand , M. E. , & Prabhakaran , V. ( 2013 ). The effect of scan length on the reliability of resting-state fMRI connectivity estimates . NeuroImage , 83 , 550 – 558 . 10.1016/j.neuroimage.2013.05.099 23747458 PMC4104183

[b7] Blondel , V. D. , Guillaume , J.-L. , Lambiotte , R. , & Lefebvre , E. ( 2008 ). Fast unfolding of communities in large networks . Journal of Statistical Mechanics: Theory and Experiment , 2008 ( 10 ), P10008 . 10.1088/1742-5468/2008/10/p10008

[b8] Buxton , R. B. ( 2013 ). The physics of functional magnetic resonance imaging (fMRI) . Reports on Progress in Physics , 76 ( 9 ), 096601 . 10.1088/0034-4885/76/9/096601 24006360 PMC4376284

[b9] Cai , B. , Zhang , G. , Zhang , A. , Xiao , L. , Hu , W. , Stephen , J. M. , Wilson , T. W. , Calhoun , V. D. , & Wang , Y.-P. ( 2021 ). Functional connectome fingerprinting: Identifying individuals and predicting cognitive functions via autoencoder . Human Brain Mapping , 42 ( 9 ), 2691 – 2705 . 10.1002/hbm.25394 33835637 PMC8127140

[b10] Calhoun , V. D. , & Adali , T. ( 2016 ). Time-varying brain connectivity in fMRI data: Whole-brain data-driven approaches for capturing and characterizing dynamic states . IEEE Signal Processing Magazine , 33 ( 3 ), 52 – 66 . 10.1109/msp.2015.2478915

[b11] Chang , C. , & Glover , G. H. ( 2010 ). Time–frequency dynamics of resting-state brain connectivity measured with fMRI . NeuroImage , 50 ( 1 ), 81 – 98 . 10.1016/j.neuroimage.2009.12.011 20006716 PMC2827259

[b12] Chen , J. , Müller , V. I. , Dukart , J. , Hoffstaedter , F. , Baker , J. T. , Holmes , A. J. , Vatansever , D. , Nickl-Jockschat , T. , Liu , X. , Derntl , B. , Kogler , L. , Jardri , R. , Gruber , O. , Aleman , A. , Sommer , I. E. , Eickhoff , S. B. , & Patil , K. R. ( 2021 ). Intrinsic connectivity patterns of task-defined brain networks allow individual prediction of cognitive symptom dimension of schizophrenia and are linked to molecular architecture . Biological Psychiatry , 89 ( 3 ), 308 – 319 . 10.1016/j.biopsych.2020.09.024 33357631 PMC7770333

[b13] Chen , J. , Tam , A. , Kebets , V. , Orban , C. , Ooi , L. Q. R. , Asplund , C. L. , Marek , S. , Dosenbach , N. U. F. , Eickhoff , S. B. , Bzdok , D. , Holmes , A. J. , & Yeo , B. T. T. ( 2022 ). Shared and unique brain network features predict cognitive, personality, and mental health scores in the ABCD study . Nature Communications , 13 , 2217 . 10.1038/s41467-022-29766-8 PMC9038754 35468875

[b14] Cho , J. W. , Korchmaros , A. , Vogelstein , J. T. , Milham , M. P. , & Xu , T. ( 2021 ). Impact of concatenating fMRI data on reliability for functional connectomics . NeuroImage , 226 , 117549 . 10.1016/j.neuroimage.2020.117549 33248255 PMC7983579

[b15] Chumin , E. J. , Cutts , S. A. , Risacher , S. L. , Apostolova , L. G. , Farlow , M. R. , McDonald , B. C. , Wu , Y.-C. , Betzel , R. , Saykin , A. J. , & Sporns , O. ( 2024 ). Edge time series components of functional connectivity and cognitive function in Alzheimer’s disease . Brain Imaging and Behavior , 18 , 243 – 255 . 10.1007/s11682-023-00822-1 38008852 PMC10844434

[b16] Cohen , J. R. ( 2018 ). The behavioral and cognitive relevance of time-varying, dynamic changes in functional connectivity . NeuroImage , 180 , 515 – 525 . 10.1016/j.neuroimage.2017.09.036 28942061 PMC6056319

[b17] Cole , M. W. , Ito , T. , Cocuzza , C. , & Sanchez-Romero , R. ( 2021 ). The functional relevance of task-state functional connectivity . Journal of Neuroscience , 41 ( 12 ), 2684 – 2702 . 10.1523/JNEUROSCI.1713-20.2021 33542083 PMC8018740

[b18] Cui , Z. , & Gong , G. ( 2018 ). The effect of machine learning regression algorithms and sample size on individualized behavioral prediction with functional connectivity features . NeuroImage , 178 , 622 – 637 . 10.1016/j.neuroimage.2018.06.001 29870817

[b19] Cutts , S. A. , Faskowitz , J. , Betzel , R. F. , & Sporns , O. ( 2023 ). Uncovering individual differences in fine-scale dynamics of functional connectivity . Cerebral Cortex , 33 ( 5 ), 2375 – 2394 . 10.1093/cercor/bhac214 35690591 PMC12954447

[b20] DeYoung , C. G. , Sassenberg , T. A. , Abend , R. , Allen , T. A. , Beaty , R. E. , Bellgrove , M. A. , Blain , S. D. , Bzdok , D. , Chavez , R. S. , Engel , S. A. , Feilong , M. , Fornito , A. , Genç , E. , Goghari , V. , Grazioplene , R. G. , Hanson , J. L. , Haxby , J. V. , Hilger , K. , Homan , P. , … Wacker , J. ( 2022 ). Reproducible between-person brain-behavior associations do not always require thousands of individuals . PsyArXiv [preprint] . 10.31234/osf.io/sfnmk

[b21] Dhamala , E. , Jamison , K. W. , Jaywant , A. , Dennis , S. , & Kuceyeski , A. ( 2021 ). Distinct functional and structural connections predict crystallised and fluid cognition in healthy adults . Human Brain Mapping , 42 ( 10 ), 3102 – 3118 . 10.1002/hbm.25420 33830577 PMC8193532

[b22] Dizaji , A. S. , Hebling Vieira , B., Khodaei , M. R. , Ashrafi , M. , Parham , E. , Hosseinzadeh , G. A. , Salmon , C. E. G. , & Soltanianzadeh , H. ( 2021 ). Linking brain biology to intellectual endowment: A review on the associations of human intelligence with neuroimaging data . Basic and Clinical Neuroscience , 12 ( 1 ), 1 – 28 . 10.32598/bcn.12.1.574.1 33995924 PMC8114859

[b23] Dubois , J. , Galdi , P. , Paul , L. K. , & Adolphs , R. ( 2018 ). A distributed brain network predicts general intelligence from resting-state human neuroimaging data . Philosophical Transactions of the Royal Society B: Biological Sciences , 373 ( 1756 ), 20170284 . 10.1098/rstb.2017.0284 PMC6107566 30104429

[b24] Eichenbaum , A. , Pappas , I. , Lurie , D. , Cohen , J. R. , & D’Esposito , M. ( 2021 ). Differential contributions of static and time-varying functional connectivity to human behavior . Network Neuroscience , 5 ( 1 ), 145 – 165 . 10.1162/netn_a_00172 33688610 PMC7935045

[b25] Elliott , M. L. , Knodt , A. R. , Cooke , M. , Kim , M. J. , Melzer , T. R. , Keenan , R. , Ireland , D. , Ramrakha , S. , Poulton , R. , Caspi , A. , Moffitt , T. E. , & Hariri , A. R. ( 2019 ). General functional connectivity: Shared features of resting-state and task fMRI drive reliable and heritable individual differences in functional brain networks . NeuroImage , 189 , 516 – 532 . 10.1016/j.neuroimage.2019.01.068 30708106 PMC6462481

[b26] Esfahlani , F. Z. , Jo , Y. , Faskowitz , J. , Byrge , L. , Kennedy , D. P. , Sporns , O. , & Betzel , R. F. ( 2020 ). High-amplitude cofluctuations in cortical activity drive functional connectivity . Proceedings of the National Academy of Sciences of the United States of America , 117 ( 45 ), 28393 – 28401 . 10.1073/pnas.2005531117 33093200 PMC7668041

[b27] Esfahlani , F. Z. , Jo , Y. , Puxeddu , M. G. , Merritt , H. , Tanner , J. C. , Greenwell , S. , Patel , R. , Faskowitz , J. , & Betzel , R. F. ( 2021 ). Modularity maximization as a flexible and generic framework for brain network exploratory analysis . NeuroImage , 244 , 118607 . 10.1016/j.neuroimage.2021.118607 34607022

[b28] Faskowitz , J. , Esfahlani , F. Z. , Jo , Y. , Sporns , O. , & Betzel , R. F. ( 2020 ). Edge-centric functional network representations of human cerebral cortex reveal overlapping system-level architecture . Nature Neuroscience , 23 ( 12 ), 1644 – 1654 . 10.1038/s41593-020-00719-y 33077948

[b29] Finn , E. S. , Shen , X. , Scheinost , D. , Rosenberg , M. D. , Huang , J. , Chun , M. M. , Papademetris , X. , & Constable , R. T. ( 2015 ). Functional connectome fingerprinting: Identifying individuals using patterns of brain connectivity . Nature Neuroscience , 18 ( 11 ), 1664 – 1671 . 10.1038/nn.4135 26457551 PMC5008686

[b30] Fong , A. H. C. , Yoo , K. , Rosenberg , M. D. , Zhang , S. , Li , C.-S. R. , Scheinost , D. , Constable , R. T. , & Chun , M. M. ( 2019 ). Dynamic functional connectivity during task performance and rest predicts individual differences in attention across studies . NeuroImage , 188 , 14 – 25 . 10.1016/j.neuroimage.2018.11.057 30521950 PMC6401236

[b31] Glasser , M. F. , Sotiropoulos , S. N. , Wilson , J. A. , Coalson , T. S. , Fischl , B. , Andersson , J. L. , Xu , J. , Jbabdi , S. , Webster , M. , Polimeni , J. R. , Van Essen , D. C. , Jenkinson , M. , & WU-Minn HCP Consortium ( 2013 ). The minimal preprocessing pipelines for the Human Connectome Project . NeuroImage , 80 , 105 – 124 . 10.1016/j.neuroimage.2013.04.127 23668970 PMC3720813

[b32] Gratton , C. , Kraus , B. T. , Greene , D. J. , Gordon , E. M. , Laumann , T. O. , Nelson , S. M. , Dosenbach , N. U. F. , & Petersen , S. E. ( 2020 ). Defining individual-specific functional neuroanatomy for precision psychiatry . Biological Psychiatry , 88 ( 1 ), 28 – 39 . 10.1016/j.biopsych.2019.10.026 31916942 PMC7203002

[b33] He , T. , An , L. , Chen , P. , Chen , J. , Feng , J. , Bzdok , D. , Holmes , A. J. , Eickhoff , S. B. , & Yeo , B. T. T. ( 2022 ). Meta-matching as a simple framework to translate phenotypic predictive models from big to small data . Nature Neuroscience , 25 ( 6 ), 795 – 804 . 10.1038/s41593-022-01059-9 35578132 PMC9202200

[b34] He , T. , Kong , R. , Holmes , A. J. , Nguyen , M. , Sabuncu , M. R. , Eickhoff , S. B. , Bzdok , D. , Feng , J. , & Yeo , B. T. T. ( 2020 ). Deep neural networks and kernel regression achieve comparable accuracies for functional connectivity prediction of behavior and demographics . NeuroImage , 206 , 116276 . 10.1016/j.neuroimage.2019.116276 31610298 PMC6984975

[b35] Heitmann , S. , & Breakspear , M. ( 2018 ). Putting the “dynamic” back into dynamic functional connectivity . Network Neuroscience , 02 ( 02 ), 150 – 174 . 10.1162/netn_a_00041 PMC6130444 30215031

[b36] Jeub , L. G. S. , Sporns , O. , & Fortunato , S. ( 2018 ). Multiresolution consensus clustering in networks . Scientific Reports , 8 , 3259 . 10.1038/s41598-018-21352-7 29459635 PMC5818657

[b37] John , Y. J. , Sawyer , K. S. , Srinivasan , K. , Müller , E. J. , Munn , B. R. , & Shine , J. M. ( 2022 ). It’s about time: Linking dynamical systems with human neuroimaging to understand the brain . Network Neuroscience , 6 ( 4 ), 960 – 979 . 10.1162/netn_a_00230 36875012 PMC9976648

[b38] Kawahara , J. , Brown , C. J. , Miller , S. P. , Booth , B. G. , Chau , V. , Grunau , R. E. , Zwicker , J. G. , & Hamarneh , G. ( 2017 ). BrainNetCNN: Convolutional neural networks for brain networks; towards predicting neurodevelopment . NeuroImage , 146 , 1038 – 1049 . 10.1016/j.neuroimage.2016.09.046 27693612

[b39] Kirkpatrick , S. , Gelatt , C. D. , & Vecchi , M. P. ( 1983 ). Optimization by Simulated Annealing . Science , 220 ( 4598 ), 671 – 680 . 10.1126/science.220.4598.671 17813860

[b40] Kong , R. , Li , J. , Orban , C. , Sabuncu , M. R. , Liu , H. , Schaefer , A. , Sun , N. , Zuo , X.-N. , Holmes , A. J. , Eickhoff , S. B. , & Yeo , B. T. T. ( 2019 ). Spatial topography of individual-specific cortical networks predicts human cognition, personality, and emotion . Cerebral Cortex , 29 ( 6 ), 2533 – 2551 . 10.1093/cercor/bhy123 29878084 PMC6519695

[b41] Kong , R. , Yang , Q. , Gordon , E. , Xue , A. , Yan , X. , Orban , C. , Zuo , X.-N. , Spreng , N. , Ge , T. , Holmes , A. , Eickhoff , S. , & Yeo , B. T. T. ( 2021 ). Individual-specific areal-level parcellations improve functional connectivity prediction of behavior . Cerebral Cortex , 31 ( 10 ), 4477 – 4500 . 10.1093/cercor/bhab101 33942058 PMC8757323

[b42] Ladwig , Z. , Seitzman , B. A. , Dworetsky , A. , Yu , Y. , Adeyemo , B. , Smith , D. M. , Petersen , S. E. , & Gratton , C. ( 2022 ). BOLD cofluctuation ‘events’ are predicted from static functional connectivity . NeuroImage , 260 , 119476 . 10.1016/j.neuroimage.2022.119476 35842100 PMC9428936

[b43] Lake , E. M. R. , Finn , E. S. , Noble , S. M. , Vanderwal , T. , Shen , X. , Rosenberg , M. D. , Spann , M. N. , Chun , M. M. , Scheinost , D. , & Constable , R. T. ( 2019 ). The functional brain organization of an individual allows prediction of measures of social abilities transdiagnostically in autism and attention-deficit/hyperactivity disorder . Biological Psychiatry , 86 ( 4 ), 315 – 326 . 10.1016/j.biopsych.2019.02.019 31010580 PMC7311928

[b44] Lake , E. M. R. , Ge , X. , Shen , X. , Herman , P. , Hyder , F. , Cardin , J. A. , Higley , M. J. , Scheinost , D. , Papademetris , X. , Crair , M. C. , & Constable , R. T. ( 2020 ). Simultaneous cortex-wide fluorescence Ca2+ imaging and whole-brain fMRI . Nature Methods , 17 ( 12 ), 1262 – 1271 . 10.1038/s41592-020-00984-6 33139894 PMC7704940

[b45] Lancichinetti , A. , & Fortunato , S. ( 2012 ). Consensus clustering in complex networks . Scientific Reports , 2 , 336 . 10.1038/srep00336 22468223 PMC3313482

[b46] Laumann , T. O. , Gordon , E. M. , Adeyemo , B. , Snyder , A. Z. , Joo , S. J. , Chen , M.-Y. , Gilmore , A. W. , McDermott , K. B. , Nelson , S. M. , Dosenbach , N. U. F. , Schlaggar , B. L. , Mumford , J. A. , Poldrack , R. A. , & Petersen , S. E. ( 2015 ). Functional system and areal organization of a highly sampled individual human brain . Neuron , 87 ( 3 ), 657 – 670 . 10.1016/j.neuron.2015.06.037 26212711 PMC4642864

[b47] Laumann , T. O. , Snyder , A. Z. , Mitra , A. , Gordon , E. M. , Gratton , C. , Adeyemo , B. , Gilmore , A. W. , Nelson , S. M. , Berg , J. J. , Greene , D. J. , McCarthy , J. E. , Tagliazucchi , E. , Laufs , H. , Schlagger , B. L. , Dosenbach , N. U. F. , & Petersen , S. E. ( 2017 ). On the stability of BOLD fMRI correlations . Cerebral Cortex , 27 ( 10 ), 4719 – 4732 . 10.1093/cercor/bhw265 27591147 PMC6248456

[b48] Leonardi , N. , & Van De Ville , D. ( 2015 ). On spurious and real fluctuations of dynamic functional connectivity during rest . NeuroImage , 104 , 430 – 436 . 10.1016/j.neuroimage.2014.09.007 25234118

[b49] Liégeois , R. , Li , J. , Kong , R. , Orban , C. , Van De Ville , D. , Ge , T. , Sabuncu , M. R. , & Yeo , B. T. T. ( 2019 ). Resting brain dynamics at different timescales capture distinct aspects of human behavior . Nature Communications , 10 ( 1 ), 2317 . 10.1038/s41467-019-10317-7 PMC6534566 31127095

[b50] Lindquist , M. A. , Xu , Y. , Nebel , M. B. , & Caffo , B. S. ( 2014 ). Evaluating dynamic bivariate correlations in resting-state fMRI: A comparison study and a new approach . NeuroImage , 101 , 531 – 546 . 10.1016/j.neuroimage.2014.06.052 24993894 PMC4165690

[b51] Liu , S. , Abdellaoui , A. , Verweij , K. J. H. , & van Wingen , G. A. ( 2023 ). Replicable brain–phenotype associations require large-scale neuroimaging data . Nature Human Behaviour , 7 ( 8 ), 1344 – 1356 . 10.1038/s41562-023-01642-5 37365408

[b52] Liu , X. , Zhang , N. , Chang , C. , & Duyn , J. H. ( 2018 ). Co-activation patterns in resting-state fMRI signals . NeuroImage , 180 , 485 – 494 . 10.1016/j.neuroimage.2018.01.041 29355767 PMC6082734

[b53] Logothetis , N. K. ( 2002 ). The neural basis of the blood–oxygen–level–dependent functional magnetic resonance imaging signal . Philosophical Transactions of the Royal Society B: Biological Sciences , 357 ( 1424 ), 1003 – 1037 . 10.1098/rstb.2002.1114 PMC1693017 12217171

[b54] Lurie , D. J. , Kessler , D. , Bassett , D. S. , Betzel , R. F. , Breakspear , M. , Kheilholz , S. , Kucyi , A. , Liégeois , R. , Lindquist , M. A. , McIntosh , A. R. , Poldrack , R. A. , Shine , J. M. , Thompson , W. H. , Bielczyk , N. Z. , Douw , L. , Kraft , D. , Miller , R. L. , Muthuraman , M. , Pasquini , L. , … Calhoun , V. D. ( 2020 ). Questions and controversies in the study of time-varying functional connectivity in resting fMRI . Network Neuroscience , 4 ( 1 ), 30 – 69 . 10.1162/netn_a_00116 32043043 PMC7006871

[b55] Marek , S. , Tervo-Clemmens , B. , Calabro , F. J. , Montez , D. F. , Kay , B. P. , Hatoum , A. S. , Donohue , M. R. , Foran , W. , Miller , R. L. , Hendrickson , T. J. , Malone , S. M. , Kandala , S. , Feczko , E. , Miranda-Dominguez , O. , Graham , A. M. , Earl , E. A. , Perrone , A. J. , Cordova , M. , Doyle , O. , … Dosenbach , N. U. F. ( 2022 ). Reproducible brain-wide association studies require thousands of individuals . Nature , 603 , 654 – 660 . 10.1038/s41586-022-04492-9 35296861 PMC8991999

[b56] Matsui , T. , Murakami , T. , & Ohki , K. ( 2019 ). Neuronal origin of the temporal dynamics of spontaneous BOLD activity correlation . Cerebral Cortex , 29 ( 4 ), 1496 – 1508 . 10.1093/cercor/bhy045 29522092

[b57] Meilă , M. ( 2007 ). Comparing clusterings—An information based distance . Journal of Multivariate Analysis , 98 ( 5 ), 873 – 895 . 10.1016/j.jmva.2006.11.013

[b58] Metropolis , N. , Rosenbluth , A. W. , Rosenbluth , M. N. , Teller , A. H. , & Teller , E. ( 1953 ). Equation of state calculations by fast computing machines . The Journal of Chemical Physics , 21 ( 6 ), 1087 – 1092 . 10.1063/1.1699114 41342507

[b59] Newman , M. E. J. , & Girvan , M. ( 2004 ). Finding and evaluating community structure in networks . Physical Review E , 69 ( 2 ), 026113 . 10.1103/physreve.69.026113 14995526

[b60] Noble , S. , Spann , M. N. , Tokoglu , F. , Shen , X. , Constable , R. T. , & Scheinost , D. ( 2017 ). Influences on the test–retest reliability of functional connectivity MRI and its relationship with behavioral utility . Cerebral Cortex , 27 ( 11 ), 5415 – 5429 . 10.1093/cercor/bhx230 28968754 PMC6248395

[b61] O’Connor , D. , Horien , C. , Mandino , F. , & Constable , R. T. ( 2022 ). Identifying dynamic reproducible brain states using a predictive modelling approach . bioRxiv [preprint] . 10.1101/2022.10.14.512147 PMC12319824 40800949

[b62] Ooi , L. Q. R. , Chen , J. , Zhang , S. , Kong , R. , Tam , A. , Li , J. , Dhamala , E. , Zhou , J. H. , Holmes , A. J. , & Yeo , B. T. T. ( 2022 ). Comparison of individualized behavioral predictions across anatomical, diffusion and functional connectivity MRI . NeuroImage , 263 , 119636 . 10.1016/j.neuroimage.2022.119636 36116616

[b63] Pannunzi , M. , Hindriks , R. , Bettinardi , R. G. , Wenger , E. , Lisofsky , N. , Martensson , J. , Butler , O. , Filevich , E. , Becker , M. , Lochstet , M. , Kühn , S. , & Deco , G. ( 2017 ). Resting-state fMRI correlations: From link-wise unreliability to whole brain stability . NeuroImage , 157 , 250 – 262 . 10.1016/j.neuroimage.2017.06.006 28599964

[b64] Parkes , L. , Fulcher , B. , Yücel , M. , & Fornito , A. ( 2018 ). An evaluation of the efficacy, reliability, and sensitivity of motion correction strategies for resting-state functional MRI . NeuroImage , 171 , 415 – 436 . 10.1016/j.neuroimage.2017.12.073 29278773

[b65] Parkes , L. , Satterthwaite , T. D. , & Bassett , D. S. ( 2020 ). Towards precise resting-state fMRI biomarkers in psychiatry: Synthesizing developments in transdiagnostic research, dimensional models of psychopathology, and normative neurodevelopment . Current Opinion in Neurobiology , 65 , 120 – 128 . 10.1016/j.conb.2020.10.016 33242721 PMC7770086

[b66] Pedersen , M. , Omidvarnia , A. , Zalesky , A. , & Jackson , G. D. ( 2018 ). On the relationship between instantaneous phase synchrony and correlation-based sliding windows for time-resolved fMRI connectivity analysis . NeuroImage , 181 , 85 – 94 . 10.1016/j.neuroimage.2018.06.020 29890326

[b67] Peña-Gómez , C. , Avena-Koenigsberger , A. , Sepulcre , J. , & Sporns , O. ( 2018 ). Spatiotemporal network markers of individual variability in the human functional connectome . Cerebral Cortex , 28 ( 8 ), 2922 – 2934 . 10.1093/cercor/bhx170 28981611 PMC6041986

[b68] Pervaiz , U. , Vidaurre , D. , Woolrich , M. W. , & Smith , S. M. ( 2020 ). Optimising network modelling methods for fMRI . NeuroImage , 211 , 116604 . 10.1016/j.neuroimage.2020.116604 32062083 PMC7086233

[b69] Preti , M. G. , Bolton , T. A. , & Van De Ville , D. ( 2017 ). The dynamic functional connectome: State-of-the-art and perspectives . NeuroImage , 160 , 41 – 54 . 10.1016/j.neuroimage.2016.12.061 28034766

[b70] Rosenberg , M. D. , & Finn , E. S. ( 2022 ). How to establish robust brain–behavior relationships without thousands of individuals . Nature Neuroscience , 25 ( 7 ), 835 – 837 . 10.1038/s41593-022-01110-9 35710985

[b71] Sakoğlu , Ü. , Pearlson , G. D. , Kiehl , K. A. , Wang , Y. M. , Michael , A. M. , & Calhoun , V. D. ( 2010 ). A method for evaluating dynamic functional network connectivity and task-modulation: Application to schizophrenia . Magnetic Resonance Materials in Physics, Biology and Medicine , 23 , 351 – 366 . 10.1007/s10334-010-0197-8 PMC2891285 20162320

[b72] Salimi-Khorshidi , G. , Douaud , G. , Beckmann , C. F. , Glasser , M. F. , Griffanti , L. , & Smith , S. M. ( 2014 ). Automatic denoising of functional MRI data: Combining independent component analysis and hierarchical fusion of classifiers . NeuroImage , 90 , 449 – 468 . 10.1016/j.neuroimage.2013.11.046 24389422 PMC4019210

[b73] Sasse , L. , Larabi , D. I. , Omidvarnia , A. , Jung , K. , Hoffstaedter , F. , Jocham , G. , Eickhoff , S. B. , & Patil , K. R. ( 2023 ). Intermediately synchronised brain states optimise trade-off between subject specificity and predictive capacity . Communications Biology , 6 , 705 . 10.1038/s42003-023-05073-w 37429937 PMC10333234

[b74] Satterthwaite , T. D. , Ciric , R. , Roalf , D. R. , Davatzikos , C. , Bassett , D. S. , & Wolf , D. H. ( 2019 ). Motion artifact in studies of functional connectivity: Characteristics and mitigation strategies . Human Brain Mapping , 40 ( 7 ), 2033 – 2051 . 10.1002/hbm.23665 29091315 PMC5930165

[b75] Schaefer , A. , Kong , R. , Gordon , E. M. , Laumann , T. O. , Zuo , X.-N. , Holmes , A. J. , Eickhoff , S. B. , & Yeo , B. T. T. ( 2018 ). Local-global parcellation of the human cerebral cortex from intrinsic functional connectivity MRI . Cerebral Cortex , 28 ( 9 ), 3095 – 3114 . 10.1093/cercor/bhx179 28981612 PMC6095216

[b76] Schwalm , M. , Schmid , F. , Wachsmuth , L. , Backhaus , H. , Kronfeld , A. , Jury , F. A. , Prouvot , P.-H. , Fois , C. , Albers , F. , van Alst , T. , Faber , C. , & Stroh , A. ( 2017 ). Cortex-wide BOLD fMRI activity reflects locally-recorded slow oscillation-associated calcium waves . eLife , 6 , e27602 . 10.7554/elife.27602 28914607 PMC5658067

[b77] Shen , X. , Finn , E. S. , Scheinost , D. , Rosenberg , M. D. , Chun , M. M. , Papademetris , X. , & Constable , R. T. ( 2017 ). Using connectome-based predictive modeling to predict individual behavior from brain connectivity . Nature Protocols , 12 ( 3 ), 506 – 518 . 10.1038/nprot.2016.178 28182017 PMC5526681

[b78] Sporns , O. , Faskowitz , J. , Teixeira , A. S. , Cutts , S. A. , & Betzel , R. F. ( 2021 ). Dynamic expression of brain functional systems disclosed by fine-scale analysis of edge time series . Network Neuroscience , 5 ( 2 ), 405 – 433 . 10.1162/netn_a_00182 34189371 PMC8233118

[b79] Sripada , C. , Rutherford , S. , Angstadt , M. , Thompson , W. K. , Luciana , M. , Weigard , A. , Hyde , L. H. , & Heitzeg , M. ( 2020 ). Prediction of neurocognition in youth from resting state fMRI . Molecular Psychiatry , 25 ( 12 ), 3413 – 3421 . 10.1038/s41380-019-0481-6 31427753 PMC7055722

[b80] Stewart , C. A. , Welch , V. , Plale , B. , Fox , G. , Pierce , M. , & Sterling , T. ( 2017 ) . Indiana University Pervasive Technology Institute . 10.5967/K8G44NGB

[b81] Thompson , G. J. ( 2018 ). Neural and metabolic basis of dynamic resting state fMRI . NeuroImage , 180 , 448 – 462 . 10.1016/j.neuroimage.2017.09.010 28899744 PMC5844792

[b82] Traag , V. A. , Van Dooren , P. , & Nesterov , Y. ( 2011 ). Narrow scope for resolution-limit-free community detection . Physical Review E , 84 ( 1 ), 016114 . 10.1103/physreve.84.016114 21867264

[b83] Uddin , L. Q. ( 2020 ). Bring the noise: Reconceptualizing spontaneous neural activity . Trends in Cognitive Sciences , 24 ( 9 ), 734 – 746 . 10.1016/j.tics.2020.06.003 32600967 PMC7429348

[b84] Uddin , L. Q. , Supekar , K. , Lynch , C. J. , Khouzam , A. , Phillips , J. , Feinstein , C. , Ryali , S. , & Menon , V. ( 2013 ). Salience network–based classification and prediction of symptom severity in children with autism . JAMA Psychiatry , 70 ( 8 ), 869 – 879 . 10.1001/jamapsychiatry.2013.104 23803651 PMC3951904

[b85] Van De Ville , D. , Farouj , Y. , Preti , M. G. , Liégeois , R. , & Amico , E. ( 2021 ). When makes you unique: Temporality of the human brain fingerprint . Science Advances , 7 ( 42 ). 10.1126/sciadv.abj0751 PMC8519575 34652937

[b86] Van Essen , D. C. , Smith , S. M. , Barch , D. M. , Behrens , T. E. J. , Yacoub , E. , Ugurbil , K. , & WU-Minn HCP Consortium . ( 2013 ). The WU-Minn Human Connectome Project: An overview . NeuroImage , 80 , 62 – 79 . 10.1016/j.neuroimage.2013.05.041 23684880 PMC3724347

[b87] Vidaurre , D. , Smith , S. M. , & Woolrich , M. W. ( 2017 ). Brain network dynamics are hierarchically organized in time . Proceedings of the National Academy of Sciences of the United States of America , 114 ( 48 ), 12827 – 12832 . 10.1073/pnas.1705120114 29087305 PMC5715736

[b88] Wehrheim , M. H. , Faskowitz , J. , Sporns , O. , Fiebach , C. J. , Kaschube , M. , & Hilger , K. ( 2023 ). Few temporally distributed brain connectivity states predict human cognitive abilities . NeuroImage , 277 , 120246 . 10.1016/j.neuroimage.2023.120246 37364742

[b89] Wu , J. , Li , J. , Eickhoff , S. B. , Scheinost , D. , & Genon , S. ( 2023 ). The challenges and prospects of brain-based prediction of behaviour . Nature Human Behaviour , 7 ( 8 ), 1255 – 1264 . 10.1038/s41562-023-01670-1 37524932

[b90] Yaesoubi , M. , Adalı , T. , & Calhoun , V. D. ( 2018 ). A window‐less approach for capturing time‐varying connectivity in fMRI data reveals the presence of states with variable rates of change . Human Brain Mapping , 39 ( 4 ), 1626 – 1636 . 10.1002/hbm.23939 29315982 PMC5847478

[b91] Yeo , B. T. T. , Krienen , F. M. , Sepulcre , J. , Sabuncu , M. R. , Lashkari , D. , Hollinshead , M. , Roffman , J. L. , Smoller , J. W. , Zöllei , L. , Polimeni , J. R. , Fischl , B. , Liu , H. , & Buckner , R. L. ( 2011 ). The organization of the human cerebral cortex estimated by intrinsic functional connectivity . Journal of Neurophysiology , 106 ( 3 ), 1125 – 1165 . 10.1152/jn.00338.2011 21653723 PMC3174820

[b92] Zalesky , A. , Fornito , A. , Cocchi , L. , Gollo , L. L. , & Breakspear , M. ( 2014 ). Time-resolved resting-state brain networks . Proceedings of the National Academy of Sciences of the United States of America , 111 ( 28 ), 10341 – 10346 . 10.1073/pnas.1400181111 24982140 PMC4104861

