# Supplementary material for: Temporal variability of brain–behavior relationships in fine-scale dynamics of edge time series
Source: Imaging Neurosci (Camb). 2025 Jan 23;3:imag_a_00443. doi: 10.1162/imag_a_00443 (PMC12319732; doi:10.1162/imag_a_00443)
Supplement: Supplementary Material [file imag_a_00443-supp.pdf]

Supporting Information

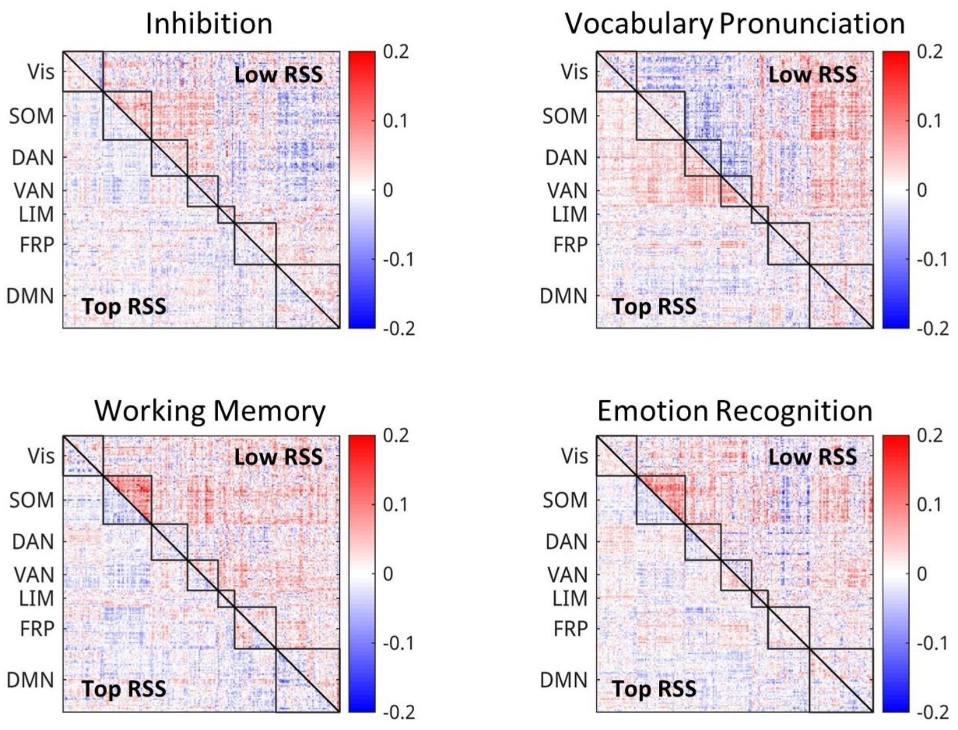

Fig. S1. Difference between brain-behavior maps of RSS deciles and full FC. Brain-behavior maps from 4 example behaviors shown in **Fig. 1B** compared to corresponding brain-behavior maps from full FC. Lower triangle shows the difference from the top RSS decile with full FC. Upper triangle displays difference from a lower RSS decile (eighth decile).

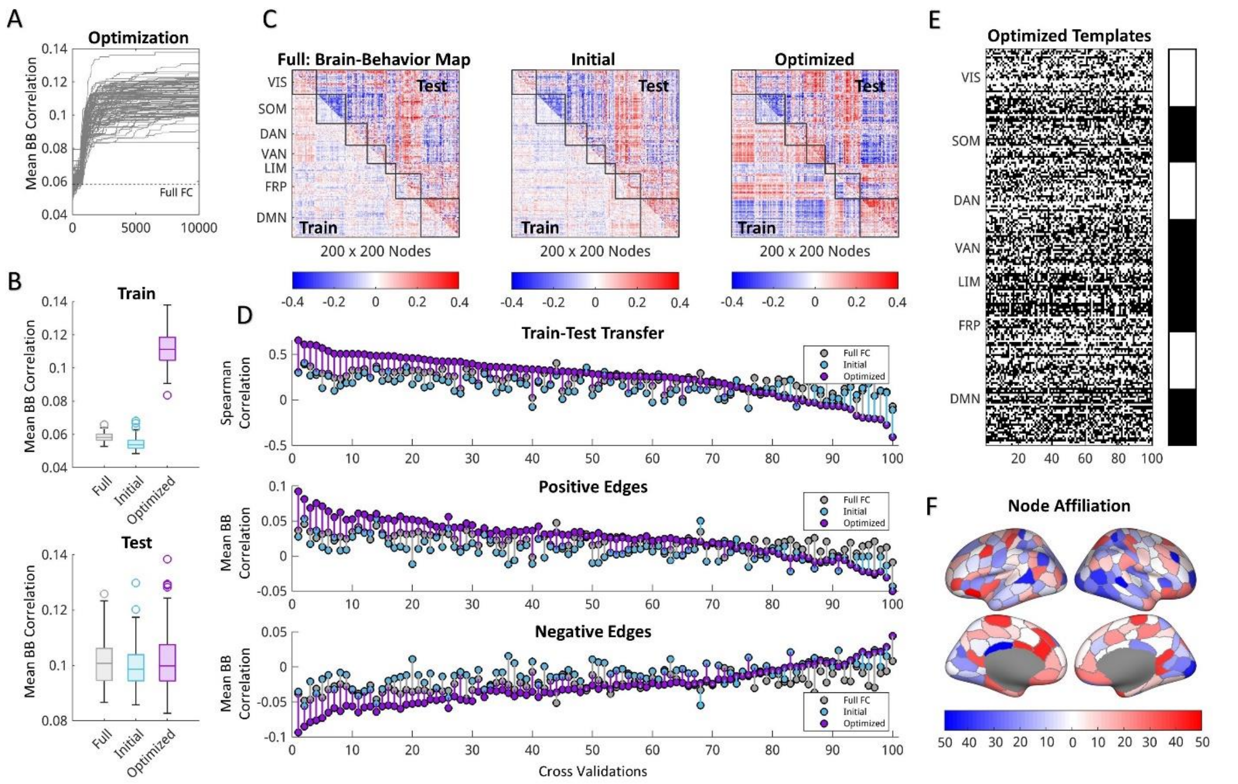

Fig. S2. Optimization results for example behavior (working memory). **(A)** Mean absolute brain-behavior correlations (mean BB) across each iteration of all cross validations compared to full FC. **(B)** Performance in mean BB for brain-behavior correlation maps created from full FC, initial templates, and optimized templates for training (*top*) and testing (*bottom*) groups across all cross validations. Boxplots display the median and interquartile range for all cross validations of vocabulary pronunciation. **(C)** Example brain-behavior correlation maps from full FC, initial, and optimized templates from best performing cross validation for training (lower diagonal) and testing (upper diagonal) subjects. **(D)** Performance of full FC, initial, and optimized templates assessed for each cross validation using Spearman correlation between training and testing brain-behavior maps (*top*) as well as mean BB of positive edges (*middle*) and negative edges (*bottom*). Positive and negative edge results are shown for the testing group using edge masks selected from brain-behavior correlation maps of training subjects and were ordered based on performance of train-test transfer (*top*). **(E)** Optimized bipartition templates for each of the 100 cross validations, rectified to align community labels across cross validations and ordered by functional systems. Templates were further down sampled to display dominant community affiliation for functional systems (*right*). **(F)** Frequency of community identities assigned to each node from E. Red and blue refer to counts of node affiliation to each template community across cross validations, where lighter colors and white refer to near chance assignments.

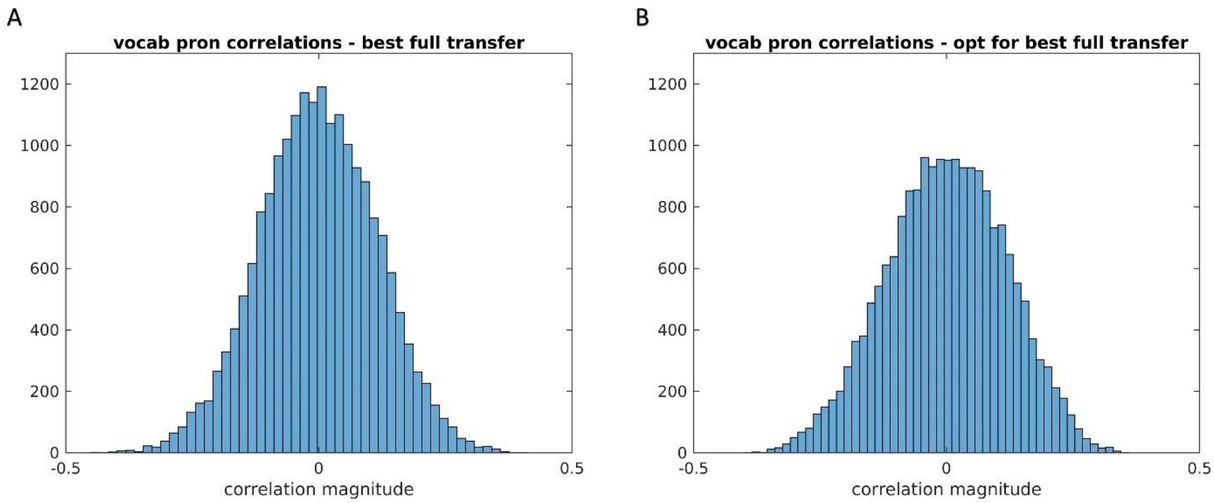

Fig. S3. Examples of brain-behavior correlation distributions for vocabulary pronunciation. Edge distributions are displayed for the testing group of the cross validation with the best train-test transfer using full FC (r = 0.39). **(A)** Edge distribution of correlations with vocabulary pronunciation using full FC. **(B)** Edge distribution of same cross validation group as A but correlations made using connectivity components from selection of optimized frames (train-test transfer r = 0.64).

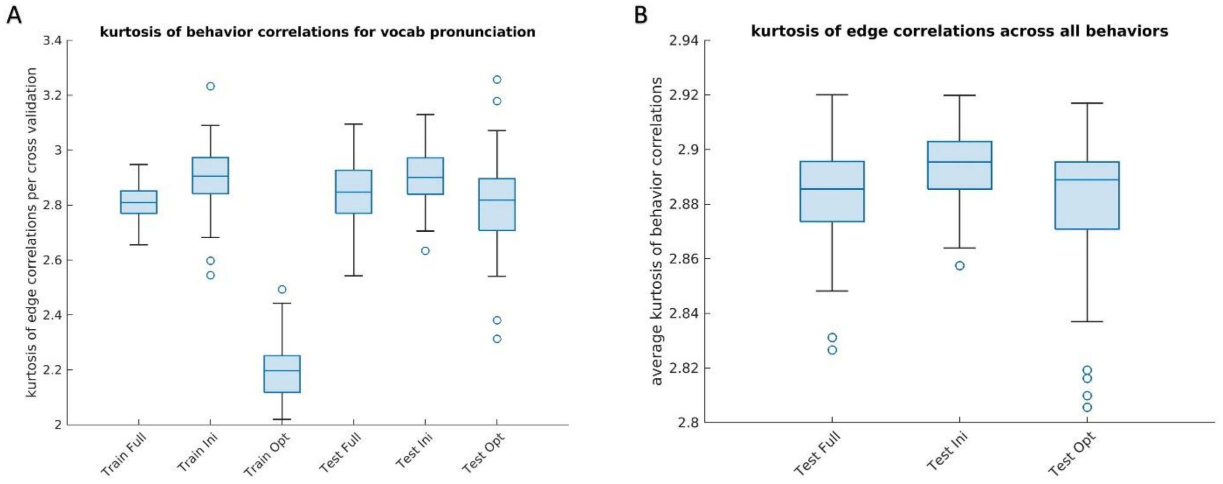

Fig. S4. Kurtosis of brain-behavior edge distributions for vocabulary pronunciation. **(A)** Kurtosis of edge correlation distributions for all cross validations using full FC, initial frames, and optimized framesets from both training and testing subject groups. In the testing group, optimized edge correlation distributions had significantly lower kurtosis than initial frames ($p < 10-8$; one-tailed paired sample t-test) and full FC ($p < 10-3$; one-tailed paired sample t-test). **(B)** Average kurtosis of edge correlation distributions for each behavior using full FC, initial, and optimized frames in the test groups. Kurtosis values were averaged across all cross validations separately for each behavior. Averaged edge correlation distributions of optimized templates had significantly lower kurtosis across all behaviors than initial templates ($p < 10-6$; one-tailed paired sample t-test) but were not significantly different than full FC.

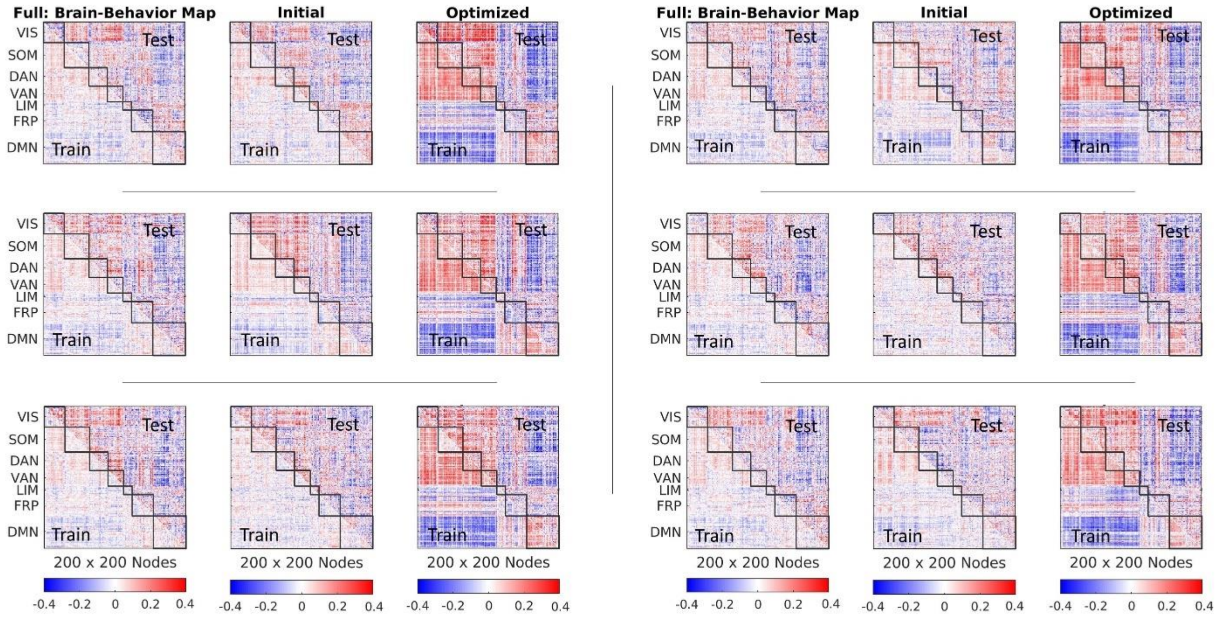

Fig. S5. Cross validation examples of brain-behavior correlation maps for vocabulary pronunciation. Results from six cross validations with the highest train-test transfer are shown for training and testing subjects from full FC, initial template, and optimized template.

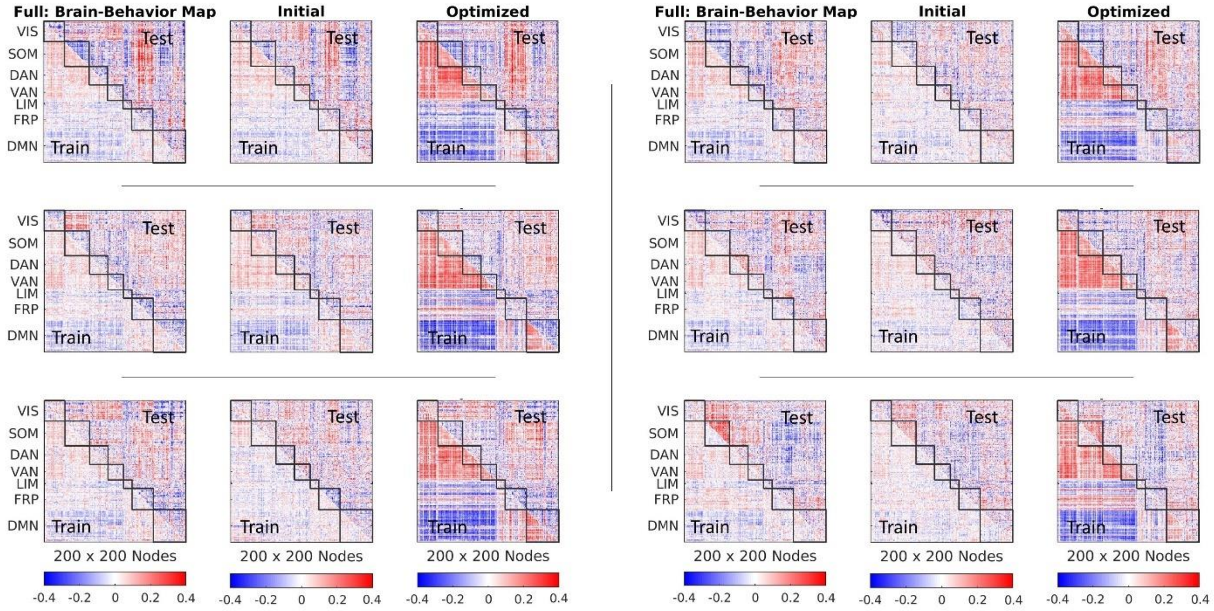

Fig. S6. Cross validation examples of brain-behavior correlation maps for vocabulary pronunciation with inverted behavioral relations. Results from six cross validations with the lowest train-test transfer are shown for training and testing subjects from full FC, initial template, and optimized template.

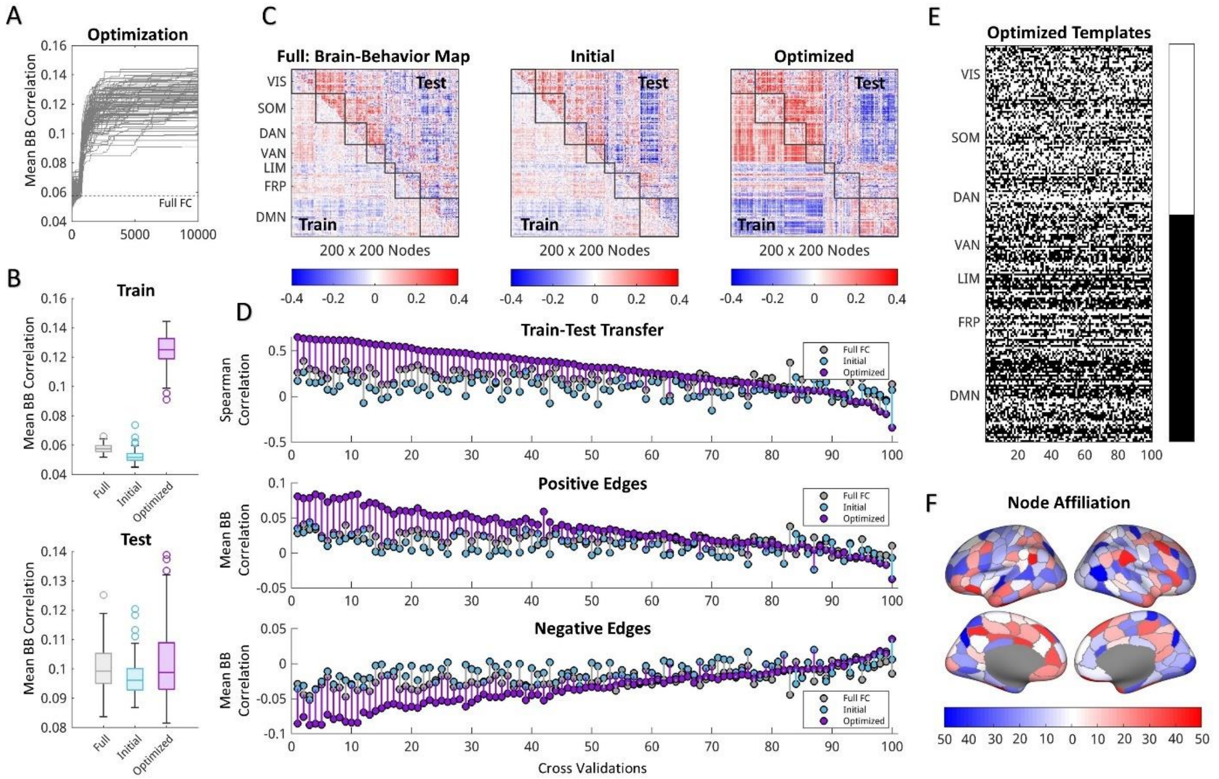

Fig. S7. Optimization results for example behavior (vocabulary pronunciation) from filtering 5% of frames. **(A)** Mean absolute brain-behavior correlations (mean BB) across each iteration of all cross validations compared to full FC. **(B)** Performance in mean BB for brain-behavior correlation maps created from full FC, initial templates, and optimized templates for training (*top*) and testing (*bottom*) groups across all cross validations. Boxplots display the median and interquartile range for all cross validations of vocabulary pronunciation. **(C)** Example brain-behavior correlation maps from full FC, initial, and optimized templates from best performing cross validation for training (lower diagonal) and testing (upper diagonal) subjects. **(D)** Performance of full FC, initial, and optimized templates assessed for each cross validation using Spearman correlation between training and testing brain-behavior maps (*top*) as well as mean BB of positive edges (*middle*) and negative edges (*bottom*). Positive and negative edge results are shown for the testing group using edge masks selected from brain-behavior correlation maps of training subjects and were ordered based on performance of train-test transfer (*top*). **(E)** Optimized bipartition templates for each of the 100 cross validations, rectified to align community labels across cross validations and ordered by functional systems. Templates were further down sampled to display dominant community affiliation for functional systems (*right*). **(F)** Frequency of community identities assigned to each node from E. Red and blue refer to counts of node affiliation to each template community across cross validations, where lighter colors and white refer to near chance assignments.

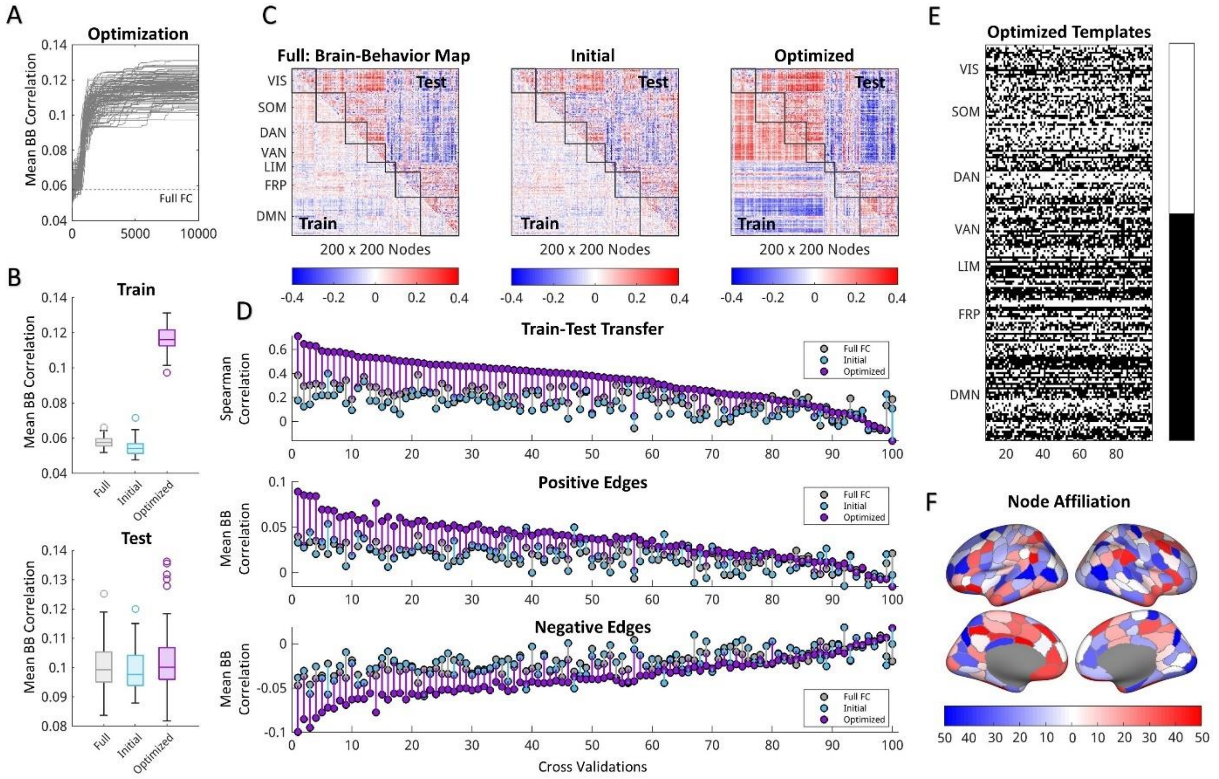

Fig. S8. Optimization results for example behavior (vocabulary pronunciation) from filtering 15% of frames. **(A)** Mean absolute brain-behavior correlations (mean BB) across each iteration of all cross validations compared to full FC. **(B)** Performance in mean BB for brain-behavior correlation maps created from full FC, initial templates, and optimized templates for training (*top*) and testing (*bottom*) groups across all cross validations. Boxplots display the median and interquartile range for all cross validations of vocabulary pronunciation. **(C)** Example brain-behavior correlation maps from full FC, initial, and optimized templates from best performing cross validation for training (lower diagonal) and testing (upper diagonal) subjects. **(D)** Performance of full FC, initial, and optimized templates assessed for each cross validation using Spearman correlation between training and testing brain-behavior maps (*top*) as well as mean BB of positive edges (*middle*) and negative edges (*bottom*). Positive and negative edge results are shown for the testing group using edge masks selected from brain-behavior correlation maps of training subjects and were ordered based on performance of train-test transfer (*top*). **(E)** Optimized bipartition templates for each of the 100 cross validations, rectified to align community labels across cross validations and ordered by functional systems. Templates were further down sampled to display dominant community affiliation for functional systems (*right*). **(F)** Frequency of community identities assigned to each node from E. Red and blue refer to counts of node affiliation to each template community across cross validations, where lighter colors and white refer to near chance assignments.

Table S1. Test results from comparisons between optimized and initial frames. Magnitude of average behavioral correlations (Mean BB) and similarity in behavioral correlation edge maps between training and testing subject groups (Transfer) were compared between AGc constructed from optimized and initial frame subsets. One-tail paired-sample t-tests were computed for Mean BB and Transfer values between cross validations using the optimized versus initial frame subsets. Significance (p-values) and effect size (Cohen's d) are displayed from these tests for each behavior. Significance of p-values is displayed with bold = p < 0.05, * = p < 0.01, and ** = p < 0.001. Effect sizes are displayed as bold = d > 0.2 (small), * = d > 0.5 (medium), and ** = d > 0.8 (large).

	Mean BB		Transfer	
**Behavior**	**p-value**	**Cohen's d**	**p-value**	**Cohen's d**
1. Visual Episodic Memory	0.2322	0.0754	**5.1×10^{-9} ** **	**0.738 ***
2. Cognitive Flexibility (DCCS)	0.8919	-0.1526	0.4641	0.0112
3. Inhibition (Flanker Task)	**4.9×10^{-5} ** **	**0.4683**	**5.7×10^{-11} ** **	**0.8841 ****
4. Fluid Intelligence (PMAT)	0.5028	-0.0008	0.0542	**0.2031**
5. Vocabulary (Pronunciation)	**9.2×10^{-7} ** **	**0.5703 ***	**3.5×10^{-21} ** **	**1.3776 ****
6. Vocabulary (Picture Matching)	0.9445	-0.1973	**0.0027 ***	**0.3399**
7. Processing Speed	0.0794	0.1325	**4.5×10^{-7} ** **	**0.6166 ***
8. Delay Discounting	0.2049	0.0921	**7.9×10^{-8} ** **	**0.5756 ***
9. Spatial Orientation	0.2452	0.0846	0.9977	-0.3573
10. Sustained Attention – Sens.	0.0522	0.1713	0.9999	-0.7104
11. Sustained Attention – Spec.	0.9758	-0.2159	0.7796	-0.0878
12. Verbal Episodic Memory	0.8686	-0.1135	0.9795	-0.2606
13. Working Memory (List Sorting)	0.183	0.1054	0.2278	0.0944
14. Cognitive Status (MMSE)	0.6294	-0.0351	0.0799	0.171
15. Sleep Quality (PSQI)	0.9229	-0.1477	0.0889	0.1518
16. Walking Endurance	**0.0055 ***	**0.2518**	**1.3×10^{-16} ** **	**0.9491 ****
17. Walking Speed	0.7116	-0.0581	**2.8×10^{-7} ** **	**0.5889 ***
18. Manual Dexterity	0.2314	0.0879	0.837	-0.1172
19. Grip Strength	0.7852	-0.0845	0.9999	-0.5198
20. Odor Identification	0.8144	-0.1059	0.8609	-0.1404
21. Pain Interference Survey	0.5469	-0.0126	0.8609	-0.1283
22. Taste Intensity	0.3647	0.0396	0.9999	-0.6145
23. Contrast Sensitivity	0.0814	0.1569	**1.7×10^{-12} ** **	**0.8551 ****
24. Emotional Face Matching	0.8881	-0.1441	0.0759	0.1743
25. Arithmetic	0.3847	0.0361	0.9999	-0.535
26. Story Comprehension	0.4674	0.0089	**2.0×10^{-11} ** **	**0.7911 ***
27. Relational Processing	0.296	0.0606	**0.0309**	**0.2278**
28. Social Cognition – Random	0.9736	-0.2285	0.9361	-0.1981
29. Social Cognition – Interaction	0.4337	0.0192	0.7301	-0.0696
30. Working Memory (N-back)	**0.0129**	**0.2063**	**9.6×10^{-8} ** **	**0.6058 ***
31. Agreeableness (NEO)	0.1596	0.1149	**0.0018 ***	**0.3908**
32. Openness (NEO)	0.6275	-0.0375	0.051	0.1974
33. Conscientiousness (NEO)	0.9687	-0.2259	0.2659	0.08
34. Neuroticism (NEO)	0.6322	-0.0344	**4.2×10^{-5} ** **	**0.5186 ***

35. Extraversion (NEO)	0.5994	-0.0294	0.8856	-0.1527
36. Emot. Recog. – Total	0.4871	0.0036	0.5891	-0.0262
37. Emot. Recog. – Angry	0.6635	-0.0487	0.9999	-0.6971
38. Emot. Recog. – Fear	0.2915	0.0615	**0.0001 ****	**0.4396**
39. Emot. Recog. – Happy	0.9476	-0.1726	0.9364	-0.1654
40. Emot. Recog. - Neutral	0.2241	0.0972	0.9994	-0.4223
41. Emot. Recog. – Sad	0.4761	0.0066	**0.0024 ***	**0.3507**
42. Anger – Affect	0.996	-0.2872	0.1191	0.1364
43. Anger – Hostility	0.9303	-0.1712	0.1501	0.1388
44. Anger – Aggression	0.985	-0.2106	**1.1×10^{-5} ****	**0.5593 ***
45. Fear – Affect	0.7462	-0.0719	0.6735	-0.0527
46. Fear – Somatic Arousal	0.0982	0.1639	0.2274	0.0872
47. Sadness	0.9276	-0.1487	0.2124	0.089
48. Life Satisfaction	0.8411	-0.1172	0.9999	-0.5697
49. Meaning & Purpose	0.8536	-0.1157	**0.0021 ***	**0.3842**
50. Positive Affect	0.2408	0.0728	0.7226	-0.0729
51. Friendship	0.8616	-0.1059	0.4268	0.0215
52. Loneliness	0.2322	0.0832	**1.9×10^{-5} ****	**0.5386 ***
53. Perceived Hostility	0.0914	0.1316	0.8175	-0.1099
54. Perceived Rejection	0.7856	-0.0962	0.1853	0.1107
55. Emotional Support	0.4948	0.0014	0.9999	-0.5947
56. Instrument Support	0.6975	-0.0491	0.9991	-0.4072
57. Perceived Stress	0.957	-0.1822	0.163	0.1149
58. Self-Efficacy	0.8917	-0.1473	0.9815	-0.2709

Table S2. Test results from comparisons between optimized and full FC. Magnitude of average behavioral correlations (Mean BB) and similarity in behavioral correlation edge maps between training and testing subject groups (Transfer) were compared between AGc constructed from optimized frame subsets and full FC. One-tail paired-sample t-tests were computed for Mean BB and Transfer values between cross validations using the optimized versus initial frame subsets. Significance (p-values) and effect size (Cohen's d) are displayed from these tests for each behavior. Significance of p-values is displayed with bold = p < 0.05, * = p < 0.01, and ** = p < 0.001. Effect sizes are displayed as bold = d > 0.2 (small), * = d > 0.5 (medium), and ** = d > 0.8 (large).

	Mean BB		Transfer	
**Behavior**	**p-value**	**Cohen's d**	**p-value**	**Cohen's d**
1. Visual Episodic Memory	0.8081	-0.0696	**0.0002 ****	**0.3889**
2. Cognitive Flexibility (DCCS)	0.825	-0.0907	0.8268	-0.1015
3. Inhibition (Flanker Task)	**1.0×10^{-5} ****	**0.4675**	**7.1×10^{-11} ****	**0.8225 ****
4. Fluid Intelligence (PMAT)	0.1651	0.1029	**0.0258**	**0.2239**
5. Vocabulary (Pronunciation)	**0.0003 ****	**0.3087**	**1.1×10^{-16} ****	**0.9011 ****
6. Vocabulary (Picture Matching)	0.9955	-0.2709	0.6373	-0.0357
7. Processing Speed	0.574	-0.013	**5.2×10^{-5} ****	**0.3557**
8. Delay Discounting	0.5941	-0.0195	**0.0013 ***	**0.2881**
9. Spatial Orientation	0.5938	-0.0231	0.9998	-0.3874
10. Sustained Attention – Sens.	**0.0003 ****	**0.2915**	0.9999	-0.4356
11. Sustained Attention – Spec.	0.9998	-0.3199	0.9606	-0.1705
12. Verbal Episodic Memory	0.9363	-0.1367	0.7433	-0.0748
13. Working Memory (List Sorting)	0.1173	0.1275	0.2383	0.078
14. Cognitive Status (MMSE)	0.6492	-0.0353	0.0817	0.141
15. Sleep Quality (PSQI)	0.9999	-0.3251	0.7503	-0.0678
16. Walking Endurance	0.3624	0.0258	**1.1×10^{-9} ****	**0.5323 ***
17. Walking Speed	0.8897	-0.094	**7.0×10^{-5} ****	**0.3357**
18. Manual Dexterity	0.1176	0.1052	0.9011	-0.1239
19. Grip Strength	0.9039	-0.1206	0.9999	-0.47
20. Odor Identification	0.6642	-0.0406	0.8785	-0.1265
21. Pain Interference Survey	0.6224	-0.0227	0.8601	-0.1167
22. Taste Intensity	**0.0179**	0.1587	0.9987	-0.3552
23. Contrast Sensitivity	0.5024	-0.0005	**1.3×10^{-9} ****	**0.6001 ***
24. Emotional Face Matching	0.8611	-0.1054	0.3872	0.0323
25. Arithmetic	0.3751	0.0313	0.9998	-0.4232
26. Story Comprehension	0.9881	-0.1784	**0.0001 ****	**0.3656**
27. Relational Processing	0.9126	-0.1199	0.2293	0.0767
28. Social Cognition – Random	0.6883	-0.0431	0.8877	-0.1446
29. Social Cognition – Interaction	0.979	-0.1792	0.9999	-0.3482
30. Working Memory (N-back)	0.2271	0.0548	**0.0078 ***	**0.2502**
31. Agreeableness (NEO)	0.186	0.0756	**0.0009 ****	**0.3696**
32. Openness (NEO)	0.4686	0.0066	0.1101	0.1337
33. Conscientiousness (NEO)	0.9978	-0.2749	0.7284	-0.0644
34. Neuroticism (NEO)	0.9752	-0.1631	**0.0027 ***	**0.2832**

35. Extraversion (NEO)	0.084	0.1156	0.8857	-0.142
36. Emot. Recog. – Total	0.1765	0.0777	0.7042	-0.0524
37. Emot. Recog. – Angry	0.9165	-0.1244	0.9999	-0.6792
38. Emot. Recog. – Fear	**0.0196**	**0.2039**	**0.0017 ***	**0.3364**
39. Emot. Recog. – Happy	0.9951	-0.2106	0.9861	-0.1898
40. Emot. Recog. - Neutral	**0.0117**	0.1794	0.979	-0.2214
41. Emot. Recog. – Sad	0.6135	-0.0254	**0.039**	0.1805
42. Anger – Affect	0.9997	-0.2794	0.5126	-0.0029
43. Anger – Hostility	0.9505	-0.1394	0.1934	0.0998
44. Anger – Aggression	0.9873	-0.1936	**4.5×10^{-7} ****	**0.5585 ***
45. Fear – Affect	0.8313	-0.0845	0.9155	-0.1339
46. Fear – Somatic Arousal	0.3615	0.0338	0.5669	-0.0164
47. Sadness	0.9273	-0.1355	0.592	-0.0235
48. Life Satisfaction	0.952	-0.1557	0.9999	-0.6037
49. Meaning & Purpose	0.9305	-0.1418	**0.0422**	**0.2068**
50. Positive Affect	0.35	0.032	0.7905	-0.0851
51. Friendship	0.7189	-0.0446	0.2507	0.0687
52. Loneliness	0.2186	0.0631	**5.2×10^{-6} ****	**0.4855**
53. Perceived Hostility	**0.0071 ***	0.1911	0.5999	-0.0255
54. Perceived Rejection	0.6529	-0.034	0.1694	0.0923
55. Emotional Support	0.5707	-0.0162	0.9999	-0.4533
56. Instrument Support	0.1353	0.0768	0.9965	-0.2901
57. Perceived Stress	0.9999	-0.3868	0.6483	-0.0364
58. Self-Efficacy	0.9581	-0.1441	0.9832	-0.2412

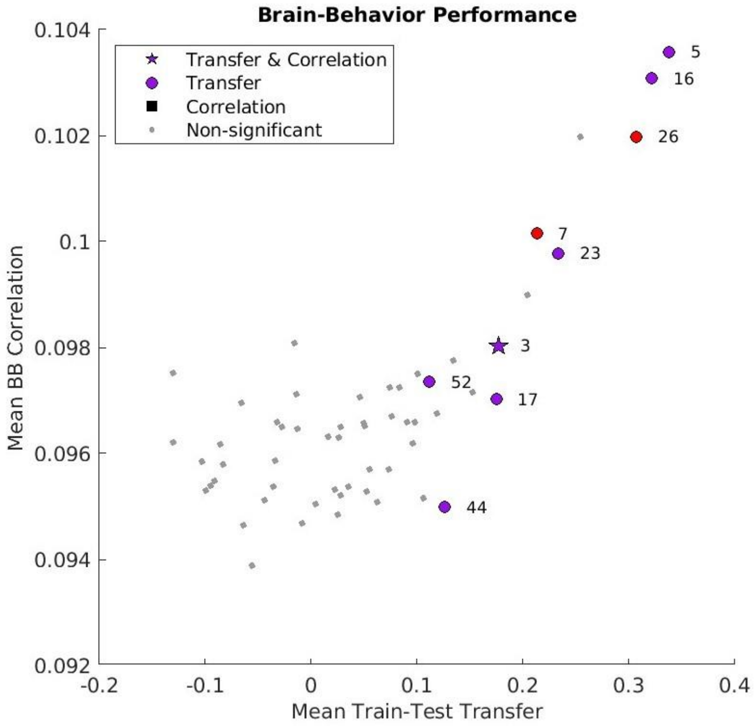

Fig. S9. Optimization summary across all behaviors Bonferroni corrected. Results from all 58 behaviors plotted as brain-behavior correlations of testing subjects versus train-test transfer (mean Spearman correlation between training and testing brain-behavior correlation maps). Across all cross validations, each behavior was compared to full FC for train-test transfer, average strength of behavioral correlations across all edges, and average strength of behavioral correlations of positive and negative edge sets. Some behaviors showed significant improvements ($p < 0.00017$, Bonferroni corrected for $\alpha = 0.01$) over full FC in all mentioned categories (star) and improvements in train-test transfer (circle). Behaviors labeled in purple additionally showed greater average strength of behavior correlations in both positive (red) and negative (blue) edges.

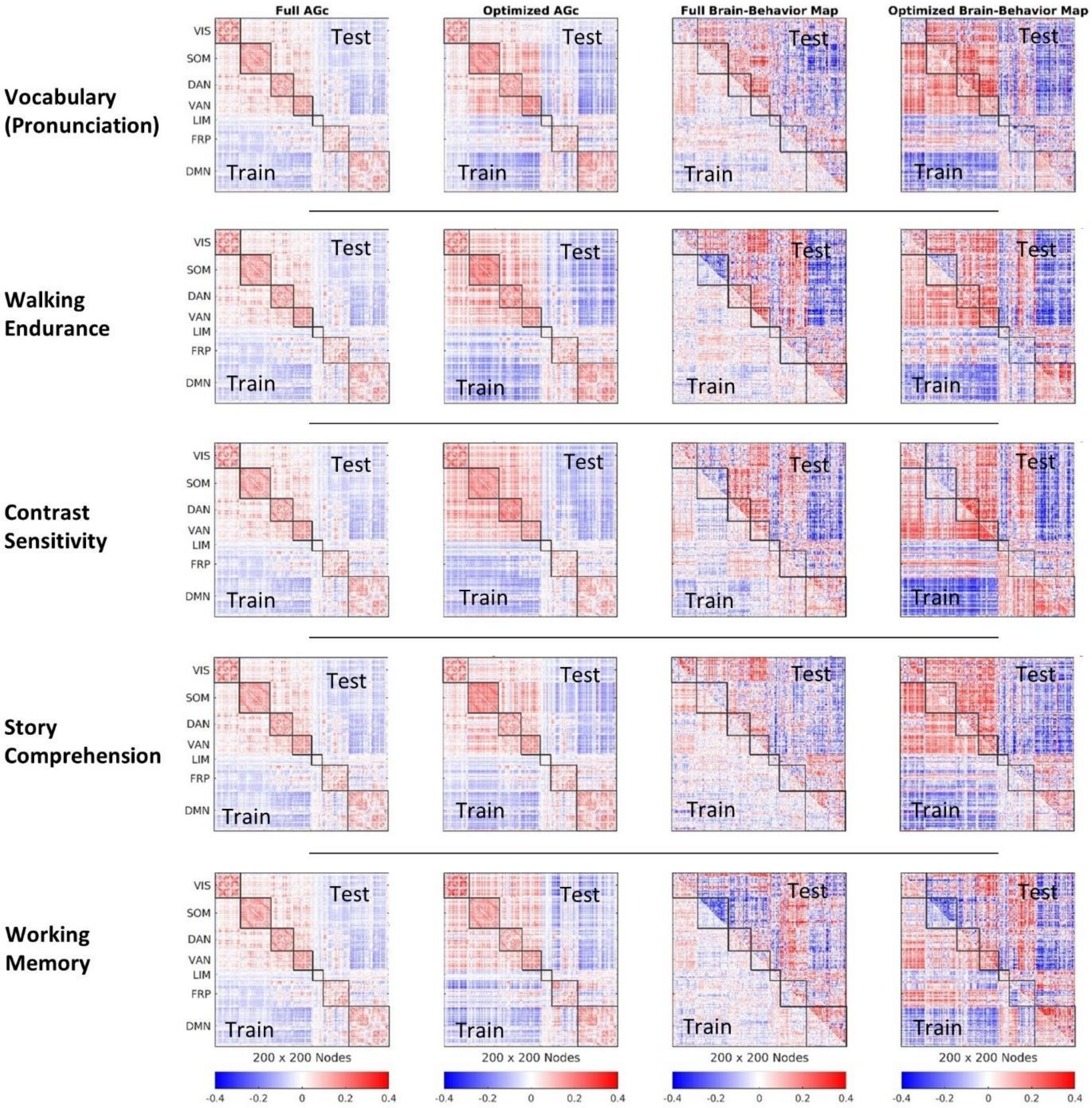

Fig. S10. Examples of filtered AGc and corresponding brain-behavior correlation maps. Examples of subject-averaged AGcs reconstructed from filtered frames and their corresponding brain-behavior correlation maps. Results from training and testing subjects are shown for the selection of all frames (Full AGc) and from the optimized template (Optimized AGc).

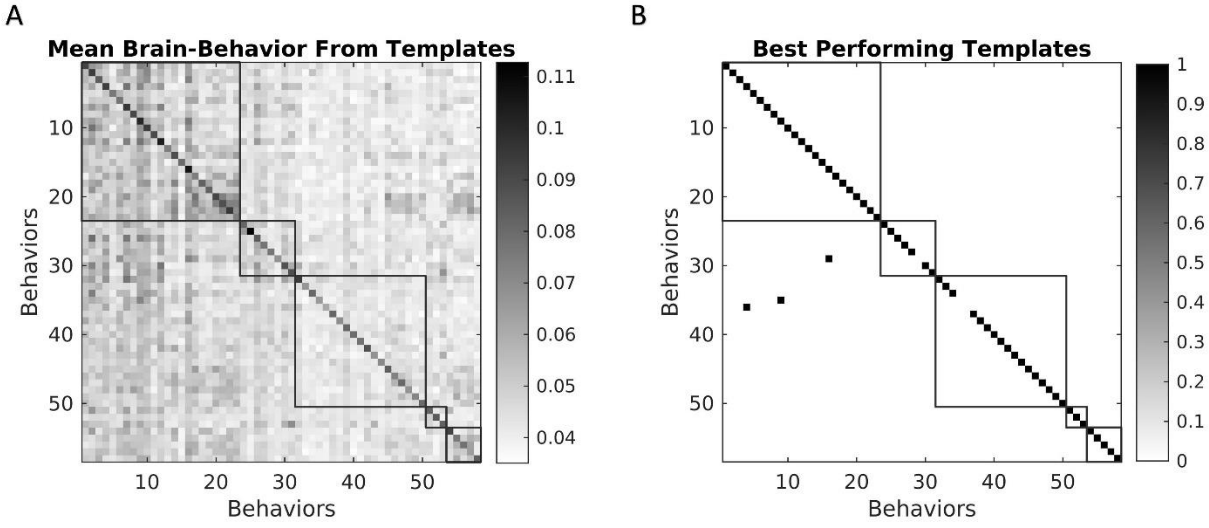

Fig. S11. Cross-behavior template performance. **(A)** Mean brain-behavior correlations from filtered frames derived separately from consensus templates. Consensus templates were computed across all cross validations of each behavior to select for the most common bipartition community assignment for each node. AGc were created from filtered frames from each consensus template and edges were correlated to each behavior. The mean brain-behavior correlations are displayed. The diagonal denotes cases where the tested and optimized behaviors match, and off-diagonal entries show performance of optimized behavior templates to different behavioral correlations. All behaviors are ordered based on the RSS clusters in Fig. 2. **(B)** Results from A but only displaying for each behavior template the corresponding behavior with the highest mean brain-behavior performance.

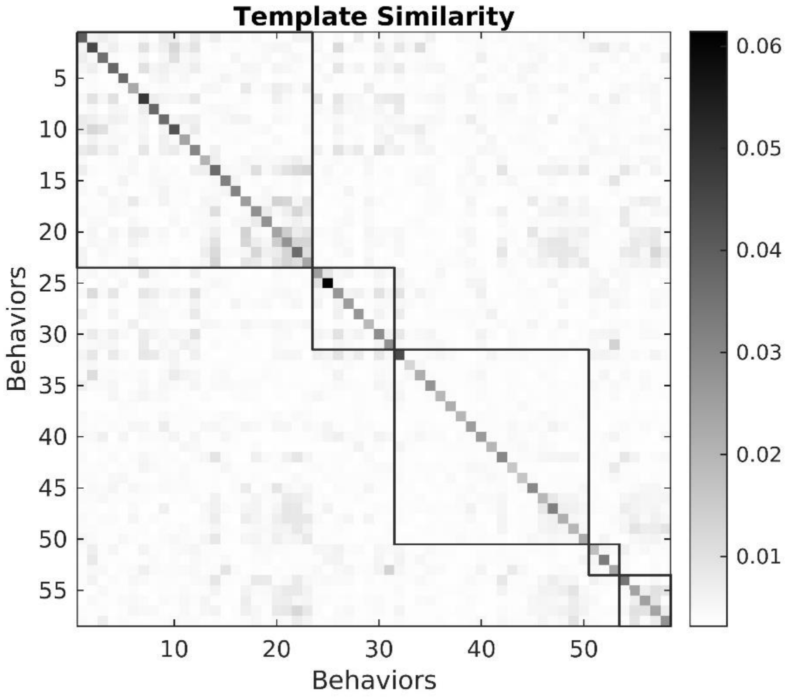

Fig. S12. Template similarity within and between behaviors. MI between optimized templates was computed between every pair of cross validations. Results are reported for MI averaged across cross validations pairs. Diagonal values display similarity of templates across cross validations within behaviors. Off-diagonal values show similarity between templates across behaviors. Behaviors are ordered based on RSS clusters from Fig 2.

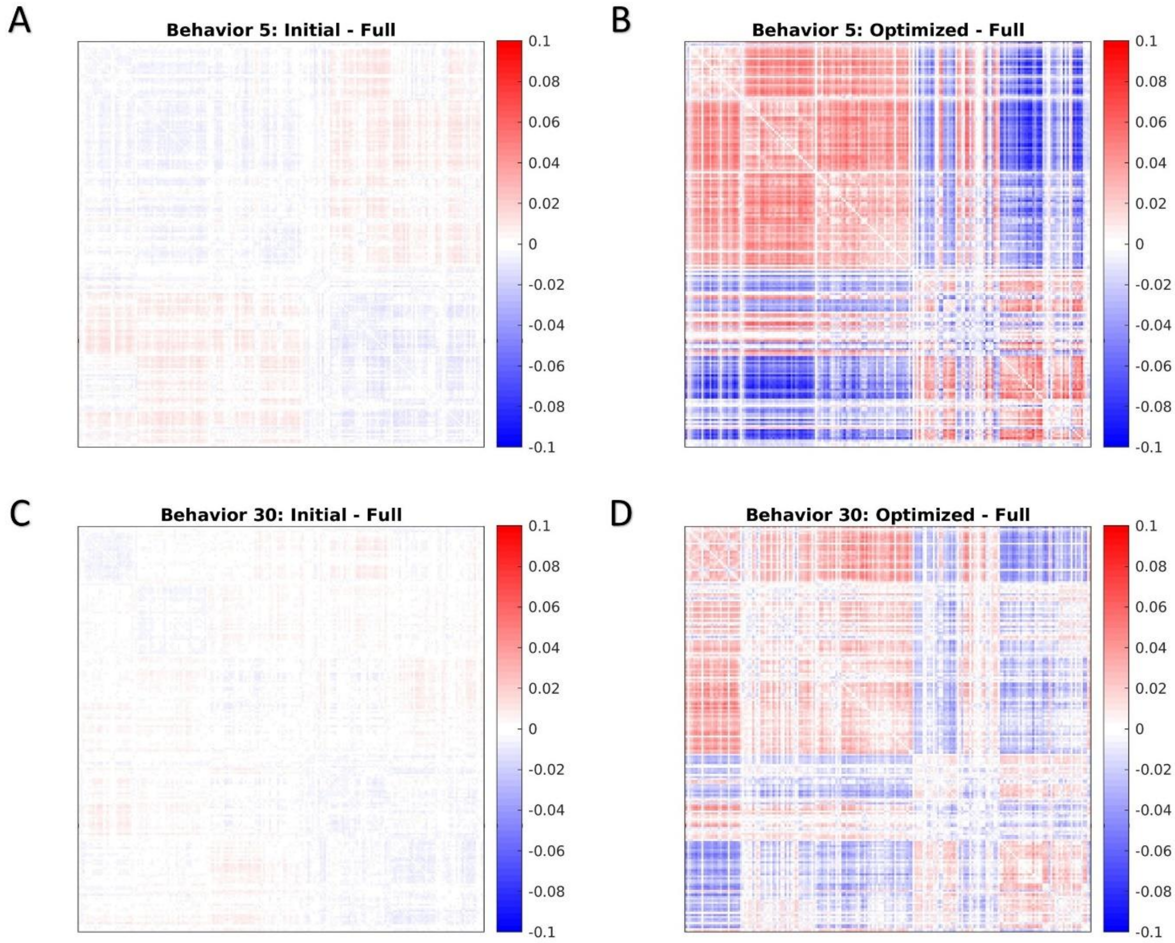

Fig. S13. Difference between filtered AGc and full FC. **(A)** Difference between AGc from the initial template and full FC averaged across testing subjects for vocabulary pronunciation (behavior 5). **(B)** Difference between AGc from the optimized template and full FC averaged across testing subjects for vocabulary pronunciation (behavior 5). **(C, D)** Results from working memory (behavior 30) for the initial template **(C)** and optimized template **(D)**.

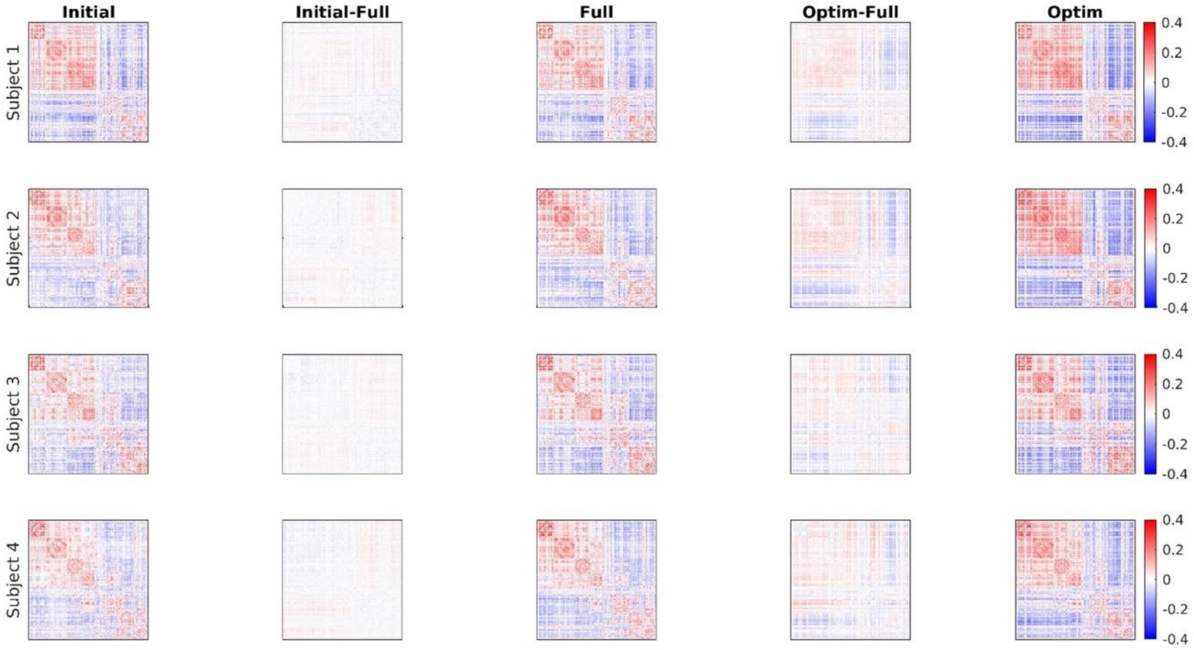

Fig. S14. Difference between filtered AGc and full FC examples from individual subjects (behavior 5 – vocabulary pronunciation). Example subject AGcs are shown for frames filtered using the initial template, all frames (Full), and optimized template (Optim). Difference maps between AGc made from initial and optimized with Full are displayed separately for each example testing subject.

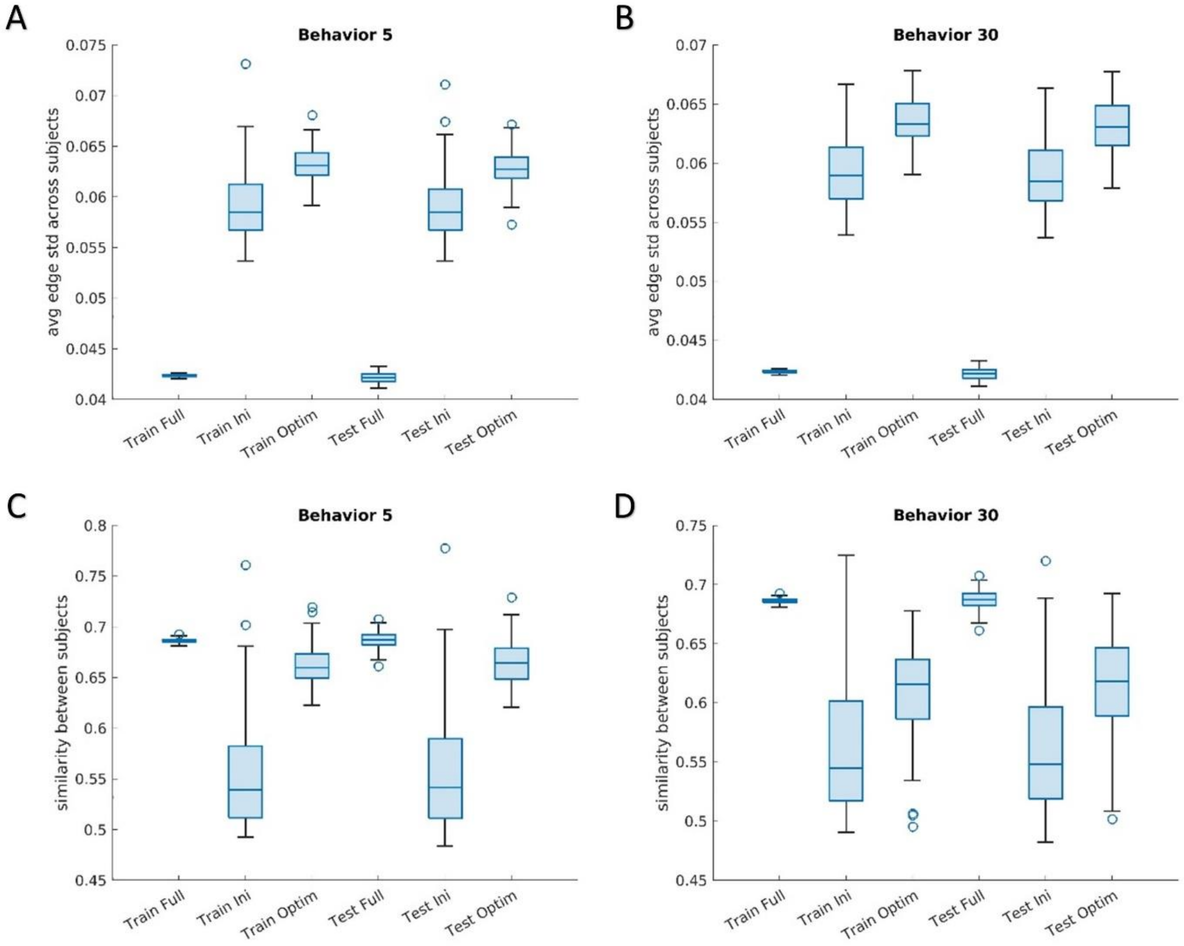

Fig. S15. Subject variability of filtered frames. **(A, B)** Average edge standard deviation across subjects for AGc made from filtered frames using full FC, initial, and filtered templates on training and testing subject groups. **(A)** Results for vocabulary pronunciation (behavior 5) show significantly greater edge standard deviation of AGc between initial and optimized as well as full FC and optimized for both the training and testing sets ($p < 10^{-16}$, paired-sample t-tests). **(B)** Results for working memory (behavior 30; all tests $p < 10^{-16}$). **(C, D)** Average similarity (Spearman correlation) of AGc between subjects for full FC, initial template, and optimized in training and testing groups for vocabulary pronunciation **(C)** and working memory **(D)**. Values of subject similarity between initial and optimized as well as full FC and optimized for both the training and testing sets were significantly different for each behavior ($p < 10^{-10}$, paired-sample t-tests). Boxplots display the median and interquartile range for averaged edge variability or averaged subject AGc similarity of each cross validation.

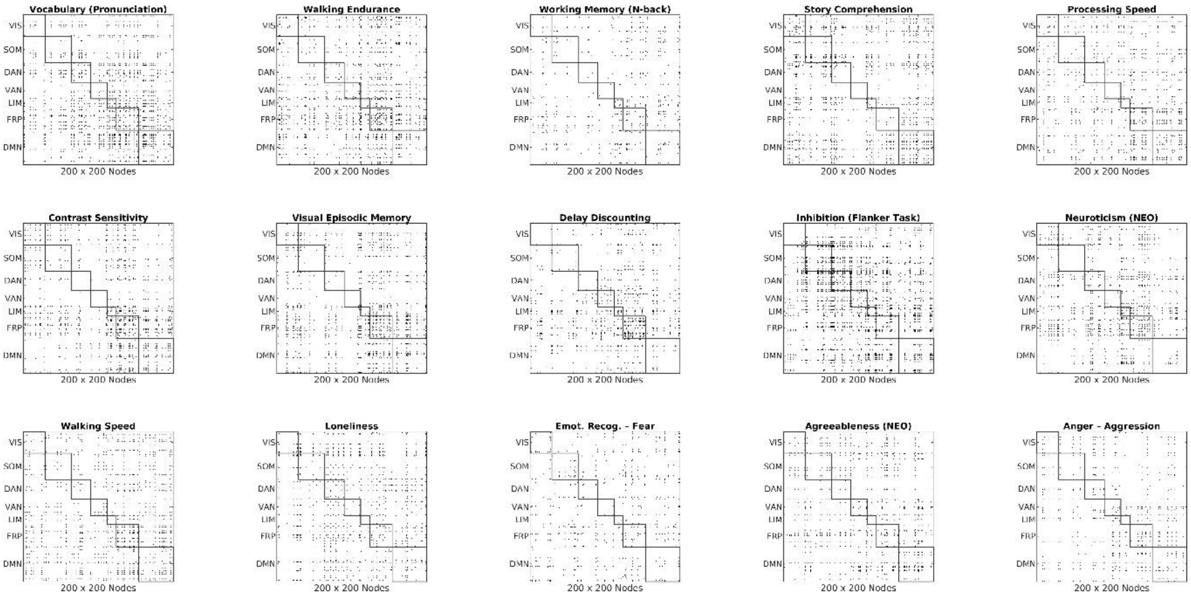

Fig. S16. Template co-assignment across node pairs. Agreement was computed across all cross validation templates of each behavior with significant improvements in behavioral correlations (**Fig. 5B**). Results are shown after thresholding the agreement matrix to only show node pairs present in at least 75% of optimizations. Functional system boundaries for the 7 Yeo networks are displayed for comparison with nodal co-assignment across templates.

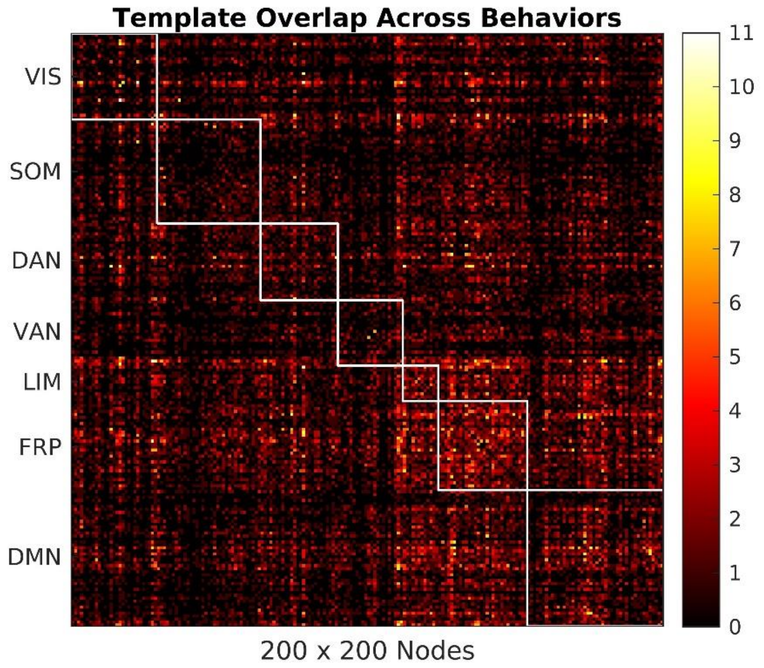

Fig. S17. Heatmap of template co-assignment matrices across behaviors. Results from **Fig. S16** were binarized and displayed here as a heatmap showing the number of behaviors (out of 15) that selected for the same co-assignment node pairings in their template structure.

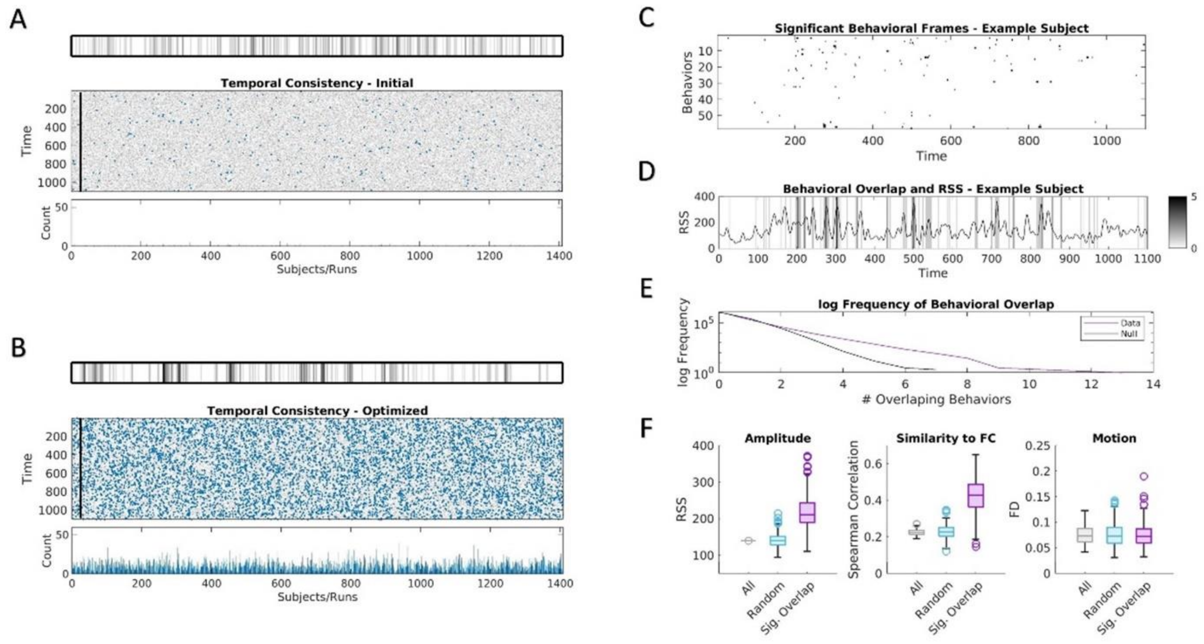

Fig. S18. Temporal consistency and behavioral overlap using frames with significant overlap of p < 0.001. **(A)** Temporal consistency of frames filtered using the initial template of example behavior (vocabulary pronunciation) across multiple cross validations. Results are shown for cross validation training templates applied to subjects in the testing groups. Frequency of frames selected across all considered cross validations for each time point are displayed for every subject and run. Example subject shown (*top*) as plotted for every subject and run (*middle*). Time points with significant consistency between cross validations (p < 0.001; compared to 10,000 circshifted nulls) plotted in blue and the frequency of occurrence of these significant time points are shown (*bottom*). **(B)** Temporal consistency of optimized templates of example behavior (vocabulary pronunciation). **(C)** Frames with significant consistency as shown in *B* plotted for each behavior across time for one example subject and run. **(D)** Overlap across behaviors of significant time points as in *C* for the example subject and run. Corresponding RSS values displayed. **(E)** Plot of log frequency of instances of behavioral overlap of significant frames across all subjects and runs of the test group compared to distribution of 10,000 circshifted nulls. **(F)** Mean characteristics of frames across all subjects and runs for all frames, matching number of randomly selected frames, significant behavioral overlap (p < 0.001) for RSS (*left*), similarity to FC (*middle*), and motion (framewise displacement; *right*). Boxplots display the median and interquartile range for averaged framewise properties of each subject.

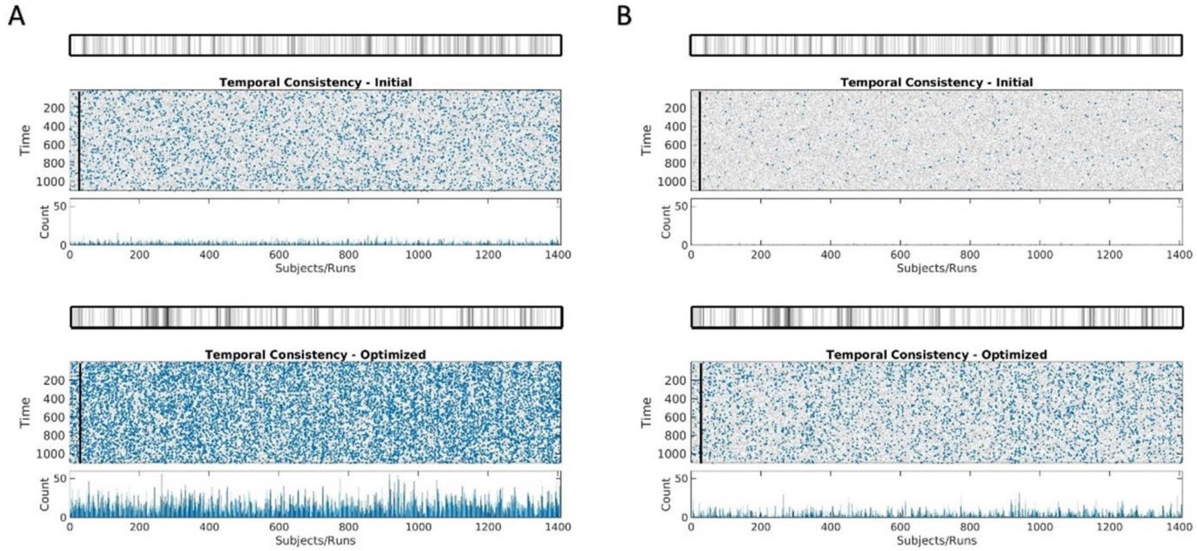

Fig. S19. Temporal consistency and behavioral overlap of working memory. **(A)** Temporal consistency of frames filtered using initial template (*top*) and optimized template (*bottom*) using a significance threshold of $p < 0.01$. **(B)** Temporal consistency of frames filtered using initial template (*top*) and optimized template (*bottom*) using a significance threshold of $p < 0.001$.
